# Genomic Analysis Reveals Distinct Concentration-Dependent Evolutionary Trajectories for Antibiotic Resistance in *Escherichia coli*

**DOI:** 10.1093/dnares/dsu032

**Published:** 2014-10-03

**Authors:** Aalap Mogre, Titas Sengupta, Reshma T. Veetil, Preethi Ravi, Aswin Sai Narain Seshasayee

**Affiliations:** National Centre for Biological Sciences, Tata Institute of Fundamental Research, GKVK, Bellary Road, Bangalore, Karnataka 560065, India

**Keywords:** antibiotic resistance, aminoglycosides, evolution

## Abstract

Evolution of bacteria under sublethal concentrations of antibiotics represents a trade-off between growth and resistance to the antibiotic. To understand this trade-off, we performed *in vitro* evolution of laboratory *Escherichia coli* under sublethal concentrations of the aminoglycoside kanamycin over short time durations. We report that fixation of less costly kanamycin-resistant mutants occurred earlier in populations growing at lower sublethal concentration of the antibiotic, compared with those growing at higher sublethal concentrations; in the latter, resistant mutants with a significant growth defect persisted longer. Using deep sequencing, we identified kanamycin resistance-conferring mutations, which were costly or not in terms of growth in the absence of the antibiotic. Multiple mutations in the C-terminal end of domain IV of the translation elongation factor EF-G provided low-cost resistance to kanamycin. Despite targeting the same or adjacent residues of the protein, these mutants differed from each other in the levels of resistance they provided. Analysis of one of these mutations showed that it has little defect in growth or in synthesis of green fluorescent protein (GFP) from an inducible plasmid in the absence of the antibiotic. A second class of mutations, recovered only during evolution in higher sublethal concentrations of the antibiotic, deleted the C-terminal end of the ATP synthase shaft. This mutation confers basal-level resistance to kanamycin while showing a strong growth defect in the absence of the antibiotic. In conclusion, the early dynamics of the development of resistance to an aminoglycoside antibiotic is dependent on the levels of stress (concentration) imposed by the antibiotic, with the evolution of less costly variants only a matter of time.

## 1. Introduction

Bacteria have an enormous potential to adapt and evolve,^1^ which is exemplified by the emergence of pathogens which are resistant to antibiotics. In this light, and with the diminishing pipeline of novel drugs, a ‘return to the pre-antibiotic era’ is almost certain unless we explore new means to counter resistance.^2^ An important step in this process is achieving a greater understanding of how antibiotics kill cells and how resistant cells counter their action.

Mechanisms of antibiotic resistance include horizontal acquisition of genes for enzymes that modify the antibiotic or its target, or transporters that export them out of the cell.^3^ For example, β-lactamases are enzymes that hydrolyze antibiotics such as penicillin. In various enteric pathogens, the transport system of AcrAB-TolC can export multiple drugs. Many antibiotics are prevalent in the natural environment, outside their role as drugs. This implies that there is a large pool of antibiotic resistance determinants in the environment.^4–7^ Additionally, mutations in the genome, which alter the target of the antibiotic or otherwise affect processes involved in antibiotic action, can also confer resistance. For example, treatment of *Escherichia coli* with the antibiotic rifampicin readily selects for mutations in its target RNA polymerase, leading to resistance.^8^ Point mutations on the genome are in fact the primary means of antibiotic resistance in *Mycobacterium tuberculosis*.^9,10^ However, since antibiotics target well-conserved and essential cellular processes, many resistance-conferring mutations in antibiotic targets might have adverse effects on fitness in the absence of the antibiotic.^11^

Aminoglycosides are a class of ribosome-targeting bactericidal antibiotics that bind the 16S rRNA of the 30S ribosomal subunit and induce mistranslation as well as translational blockade.^12^ Aminoglycosides have a streptamine or a 2-deoxystreptamine (2-DOS) ring. The commonly used antibiotic streptomycin belongs to the former class. The latter group of aminoglycosides includes antibiotics such as kanamycin, gentamicin, neomycin and paromomycin.^13^ 2-DOS aminoglycosides typically bind to the ribosomal decoding site in the 16S rRNA and cause a conformational change, which leads to the recognition of near-cognate codons and therefore miscoding.^14,15^ This interaction, along with the binding of the antibiotic to the 23S rRNA in the large subunit,^16,17^ also causes translocation blockade^15,18^ and inhibition of ribosome recycling.^16,19^ Of these mechanisms, translational miscoding might play only a minor role in cell killing, going by the evidence that strains with ribosomal mutations known to reduce translation fidelity are viable.^20–22^ This probably lays greater emphasis on the translation blockade that these antibiotics cause^23^ through their effects on ribosome translocation and recycling. Though various other non-intuitive mechanisms for cell death such as oxidative stress have been proposed, these remain controversial.^24–26^

A common method by which bacteria acquire resistance to aminoglycosides is via enzymes that modify the antibiotic, such as aminoglycoside acetyltransferases and phosphotransferases.^27^ These genes are generally used as selectable markers in routine laboratory work. In addition, mutations targeting the aminoglycoside binding site in the 16S rRNA can confer resistance.^28,29^ Spontaneous emergence of such mutants in certain bacteria confers two orders of magnitude-fold resistance to the antibiotic.^30^ However, such mutations are not accessible to *E. coli* due to the presence of multiple copies of the 16S rRNA gene,^12^ a property common to many fast-growing bacteria. Even in the event that a single copy of the gene gets mutated, sensitivity to aminoglycosides is dominant in a heterogeneous ribosomal population.^31^ Previous studies on mutations conferring resistance to the aminoglycoside kanamycin in *E. coli* have led to the isolation of two elongation factor-G (EF-G) mutations. However, besides showing a temperature-sensitive phenotype, these bear a heavy cost to the cell (by affecting translation) in the absence of the antibiotic,^32^ a property common to resistance mutations for many classes of antibiotics.^11^ A more recent large-scale screen for aminoglycoside resistance suggested that reduction of proton motive force (PMF) on the membrane confers resistance, while making the cell more sensitive to other antibiotics that require PMF for efflux.^33^

Sublethal concentrations of antibiotics might play important physiological roles in bacteria, by acting as signalling molecules affecting gene expression.^7^ Besides, these can also promote development of resistance, either by induction of specific mutator pathways^34^ or—in the natural environment—by promotion of horizontal gene transfer.^35^ More fundamentally, under low antibiotic concentrations, growth of a subpopulation of cells that do not see the antibiotic, or the uniformly slower growth of the entire cell population, provides a substrate for selection to act. For example, antibiotics at concentrations as low as 1/100th the killing concentration leads to the selection of resistant bacteria.^36^ Sublethal concentrations of antibiotics are important in a clinical context, because factors such as incorrect antibiotic dosage or non-compliance of the patient with a prescribed dose regime could lead to low levels of the antibiotic in the body. Therefore, it is important to understand the effect of antibiotic dosage in the development of resistance, and the effects of the ensuing resistance mechanisms on fitness in the absence of the antibiotic.

The development of next-generation deep-sequencing technologies has made it relatively easy to track the emergence of variants in a population, including in the context of antibiotic resistance.^37–39^ In this study, we use laboratory evolution of *E. coli* in batch cultures, followed by deep sequencing, to interrogate the emergence of resistance to an aminoglycoside antibiotic at two different sublethal concentrations. We show that resistance mechanisms with little consequence to cell fitness in the absence of the antibiotic emerge rapidly, and that the early dynamics of development of resistance might be dependent on the concentration of the antibiotic.

## 2. Materials and methods

### 2.1. Strains, culture media, growth curves and optical density

Non-pathogenic *E. coli* MG1655 was used for the evolution experiments. Growth curves were generated in flasks or 96-well plates in Luria Bertani (LB; Hi-Media, India) broth using 1:100 dilution of overnight culture and incubation at 37°C with shaking at 200 rpm. Optical density (OD) measurements were carried out at 600 nm (OD_600_) using either a UV-visible spectrophotometer (SP-8001, Metertech) when growth curves were generated in flasks or using a plate reader (Infinite F200pro, Tecan) when generated in 96-well plates.

### 2.2. Minimum inhibitory concentration determination

The minimum inhibitory concentration (MIC) was determined by a modification of the broth dilution technique. Antibiotics were obtained from Sigma-Aldrich (Kanamycin sulfate K1377, Streptomycin sulfate salt S6501, Gentamicin sulfate salt G1264, Neomycin trisulfate salt hydrate N1876, Paromomycin sulfate salt P5057, Hygromycin B H7772, Apramycin sulfate salt A2024). Dilutions of the antibiotic stock were made in sterile distilled water to which equal volume of 2× concentrated LB broth was added. Amount of stock taken was calculated with respect to final diluted volume. Overnight grown culture was added to the broth to achieve 1:100 dilution. After 24-h incubation at 37°C with shaking at 200 rpm, the MIC was inferred as the lowest concentration at which OD_600_ falls below 0.05 with respect to the blank. Eight independently grown replicates were so tested for their MIC.

### 2.3. Evolution in kanamycin

For evolution experiments in sublethal kanamycin concentrations (Supplementary Fig. S1), the sublethal window was defined on the basis of an MIC of kanamycin for *wild type* (Supplementary Fig. S2A). We selected two concentrations for the evolution experiment: 4 µg/ml (4-kan, ∼25% MIC) and 8 µg/ml (8-kan, ∼50% MIC); growth parameters like length of lag period, growth rates and stationary-phase OD_600_ were significantly different at these two concentrations (Supplementary Fig. S3). Evolution experiments were carried out in a 100-ml LB broth in 250 ml conical flasks with incubation at 37°C and shaking at 200 r.p.m. Two replicate populations in the case of 0-kan and 4-kan, and four replicate populations in the case of 8-kan, were passaged by batch culture into fresh 100-ml LB broth (while maintaining kanamycin concentration) using 1:100 dilution every 24 h, under identical shaking and incubation conditions. For the 4-kan populations, the MICs of the two replicate populations were followed with each passage, and populations were collected by spinning down the culture for genomic DNA (gDNA) extraction and deep sequencing. Glycerol stocks were also maintained for each passage. For the 8-kan populations, the MICs of colonies derived from these populations on plain LB agar were followed with each passage. A few colonies were selected and grown in LB broth for collecting cells for gDNA extraction and deep sequencing; glycerol stocks for these were also prepared.

### 2.4. Population structure analysis of evolving bacterial cultures

As described in the previous section, we grew *E. coli* batch cultures in 0-kan (plain LB), 4-kan and 8-kan up to one passage. Cultures at the 0 h (passage 0), 24 h (passage 0) and 24 h (passage 1) were subjected to serial 10-fold dilution and plated on plain LB agar plates. The resulting colonies were randomly picked and tested for resistance by growth in two different concentrations of kanamycin: 4-kan (moderately-lethal) and 20-kan (lethal), and tested for defects in growth in plain LB (0-kan). Only 24 h OD_600_ was measured. This experiment was performed on around 88 isolates obtained from each of the three evolving populations (0-kan, 4-kan and 20-kan) at the three indicated time points. We did not maintain replicate populations; instead, we repeated the experiment multiple times. Results of two different repeats of the experiment that had considerable variation are shown in Supplementary Fig. S5.

### 2.5. Genomic DNA extraction

Extraction of gDNA from either experimental populations or individual isolates from such populations was done using the Sigma-Aldrich GenElute Bacterial Genomic DNA Kit (NA2120) using the manufacturer's protocol. gDNA integrity was checked on agarose gel, and quality and concentration were checked using NanoDrop UV-Vis Spectrophotometer (Thermo Scientific).

### 2.6. Sequencing and determination of mutations

Library preparation was carried out using the Truseq DNA library sample prep kit V2, and sequencing was carried out using TruSeq SBS Kit v3-HS (50 Cycles) at Centre for Cellular and Molecular Platforms (C-CAMP) on Illumina HiSeq 1000 platform according to manufacturer's instructions. Samples were sequenced to ∼300× coverage. FASTX quality assessed reads were mapped to the reference genome (NC_000913.2) using BWA.^40^ SAMTOOLS^41^ was used to process the BWA output aligned sam files to generate a pileup file, and VARSCAN^42^ was used to extract the list of single nucleotide polymorphisms (SNPs) and indels from the pileup. Percentage of reads supporting a SNP was obtained by dividing the number of reads containing the SNP by the total number of reads mapping to that locus, multiplied by 100. In the case of 4-kan populations, mutations were called when percentage of reads supporting the mutant allele was above 20% in at least one of the sequenced time points during the course of the evolution experiment. This cut-off was set to 50% for isolate sequencing in the case of 8-kan populations; we note here that though a low cut-off was set, mutations identified in the 8-kan clones were generally supported by ∼97% of reads. Once the list of mutations was generated using these cut-offs, their abundances across all time points were recalculated from the pileup files using a perl script. Only good-quality bases (quality > phred score 30) were considered for this analysis, and bases at the start and end of reads that mapped to the mutation position were excluded. Additionally, BRESEQ^43^ was used to verify the mutations. Longer deletions in the genes encoding subunits of the ATP synthase complex were inferred by BRESEQ. Sanger sequencing was done to confirm mutations.

### 2.7. Phage transduction

To move the FusA^P610T^ mutation into the *wild type* background for characterization, phage transduction using the P1 phage was used. The kanamycin resistance cassette, amplified from pKD13, was inserted in a featureless region near the *fusA* gene (without deleting a single nucleotide) using the one-step inactivation procedure of Datsenko and Wanner.^44^ (A higher concentration of kanamycin, i.e. 80 µg/ml, was used to limit growth of the resistant parent strain.) This strain was used as donor to prepare P1 phage lysate that was subsequently used to transduce *wild type* as per the Court lab protocol.^45^ Selection for transductants was done on LB agar plates containing kanamycin. FLP recombinase provided by pCP20 was used to remove the kanamycin resistance cassette which is flanked by FRT sites (leaving behind a FRT site in the process).

### 2.8. Growth simulation

Discrete growth simulations were used to assess the effect of different growth rate regimes on the persistence of sick variants. Each simulation was seeded with *N* objects—each object representing a bacterial cell—of three types as follows. Most of these objects were *wild type*. A small number of objects represented mutants of two types, one with a higher growth rate than the other (3-fold difference in growth rates), with both mutants displaying a higher rate of growth than the *wild type* (with the *wild type* growing three times slower than the slow-growing mutant). This set-up reflects the situation wherein two resistant mutants—one with a growth defect and the other not—are competing with each other and the *wild type* for growth in the presence of sublethal concentrations of an antibiotic. The simulation was seeded with five times as many sick mutants as fit ones. Each growth iteration would allow an object to replicate or not—the probability that an object replicates would depend on the growth rate. The simulations were stopped when the total number of objects crossed a pre-defined threshold. This reflects only exponential growth. Introduction of a subsampling step after a certain number of iterations, to simulate serial passages, does not alter these results.

### 2.9. Competition assays

To distinguish between two competing MG1655 strains, a knock-out of *lacZ* gene was generated in *wild type* MG1655 background using the one-step inactivation procedure of Datsenko and Wanner.^44^ The colonies of a Δ*lacZ* strain can be distinguished from those of *lacZ^+^* colonies on McConkey's agar (HiMedia, India). The *lacZ* deletion was made in both *wild type* and *wild-type*-FRT backgrounds. For competition, *wild type*/*wild-type*-FRT (Δ*lacZ*) cultures were co-inoculated in LB along with FusA^P610T^-FRT (*lacZ^+^*) in 1:1 proportion, and the counts of both colony types were followed on LB agar. As control, mock competitions were carried out with the two (otherwise competing) cultures grown separately. They were mixed together in 1:1 proportion immediately prior to performing viable count on the differentiating medium.

### 2.10. GFP induction

Hou *et al.*^32^ assayed translation defects in their kanamycin-resistant *fusA* mutants by looking at the kinetics of induction of β-galactosidase in these strains versus the *wild type*. Similarly, we looked at the kinetics of induction of green fluorescent protein (GFP) to check for translation defects in FusA^P610T^. GFP^mut2 46^ was cloned from pMS201 into pBAD18^47^ within *Eco*RI and *Hin*dIII restriction sites to get pBAD18::*gfpmut2*. Overnight cultures of both WT-FRT and FusA^P610T^-FRT were grown in LB broth in a 96-well black plate (Sigma-Aldrich, costar 3603) until OD_600_ reached ∼0.5. Subsequently to induce GFP^mut2^, 1 or 10 mM arabinose (HiMedia, India) was added to the wells that were to be tested for induction. Also, kanamycin was added to the wells that were to be tested for induction in the presence of the antibiotic, to a final concentration of 10 μg/ml. GFP^mut2^ fluorescence was measured at 520 nm by excitation at 485 nm using a plate reader (Infinite F200pro Tecan). Promoter activities^48^ were calculated as follows: OD and GFP fluorescence intensity measurements were blank subtracted separately. GFP intensity was then normalized to OD. Promoter activity was calculated as d*F*/d*t*, where d*F* is change in OD-normalized GFP fluorescence in time interval d*t*. Finally, promoter activities thus calculated were normalized to fall in the range of 0–1.

## 3. Results

### 3.1. Growth of E. coli in sublethal concentrations of kanamycin

To explore the landscape of mutations leading to resistance of *E. coli* to aminoglycosides, we grew the bacteria—in a batch culture—in two sublethal concentrations of kanamycin (4 µg/ml, ∼20–25% of the MIC; 8 µg/ml, ∼40–50% of the MIC; see ‘Materials and Methods’; Supplementary Figs S1 and S2A), a 2-DOS aminoglycoside antibiotic commonly used in the laboratory. Here we refer to the first batch culture of bacteria in antibiotic-containing medium as P0. A sample of bacteria obtained after ∼24 h of growth in P0 was serially passaged under identical conditions, and these serial passages will be referred to as P1, P2, etc.

**Figure 1.**
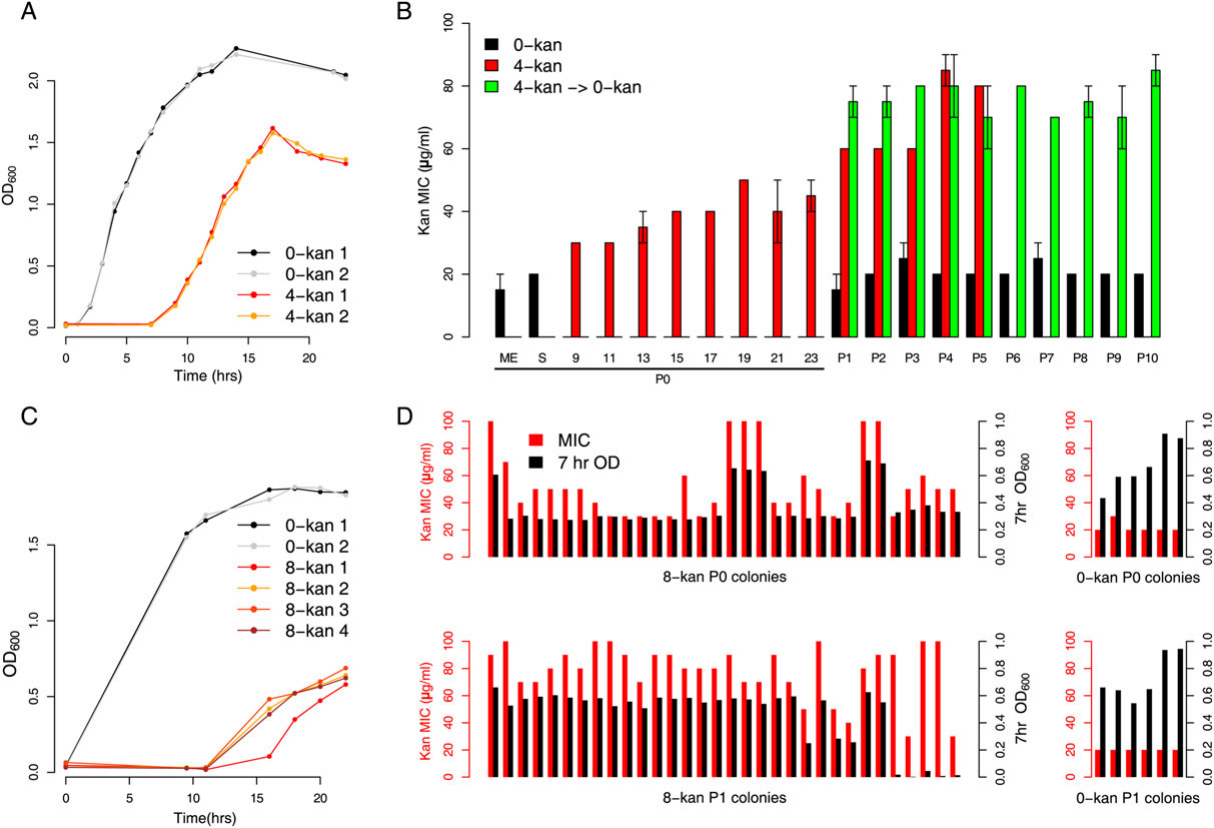
Resistance developed as a result of growth in sublethal kanamycin concentration. (A) Growth of two replicate populations of *Escherichia coli* MG1655 in ∼25% of the lethal level of kanamycin (4 µg/ml: 4-kan 1 and 2; for additional growth curves, see Supplementary Figure S3A). Control populations in plain LB are 0-kan 1 and 2. Populations at various time points from the 4-kan curves were sequenced while only mid-exponential- and stationary-phase populations from the 0-kan curves were sequenced. (B) MICs of evolving populations. P0 indicates the first round of growth in kanamycin (i.e. the growth curves in A) while P1–P10 indicate serial transfers by 100-fold dilution. Black bars indicate MIC levels of control populations passaged in plain LB (0-kan). Red bars indicate populations evolving in 4-kan, with data available for multiple time points in P0. Green bars indicate populations grown in 4-kan in P0 but subsequently transferred into plain LB for the remaining passages P1–P10 (4-kan → 0-kan). The maintenance of increased MIC levels suggests the presence of resistant mutations and not a temporary stress response. The average MIC level of the two biological replicates (from three technical replicates) is plotted with the total length of the positive and negative error bars representing the difference in MICs between the two replicates (range). (C) Growth of four replicate populations of *E. coli* MG1655 in ∼50% lethal levels of kanamycin (8 µg/ml: 8-kan 1-4; more growth curves can be found in Supplementary Figure S3). (D) Isolates derived from (four replicates of) the P0 8-kan and the P1 8-kan populations were tested for their MIC (red bars) and growth in plain LB (black bars, taken at 7 h. after inoculation). Many isolates in P0 were sick, i.e. had a lower OD_600_ in plain LB but could resist kanamycin. The incidence of these sick isolates decreases after P1, and fit resistant forms seem to dominate. For comparison, kanamycin MICs (red bars) and OD_600_ (black bars) of multiple isolates, from P0 and P1 control *wild-type* populations grown in plain LB, are shown in the right panels.

The P0 growth curve of *E. coli* grown in 4 µg/ml kanamycin (4-kan) is characterized by a long lag phase (6–7 h) followed by an exponential phase defined by low growth rate (Fig. 1A and Supplementary Fig. S3 for statistics). The lag phase period increased considerably to at least 11 h in 8 µg/ml kanamycin (8-kan) (Fig. 1C and Supplementary Fig. S3); the cultures typically reached a final OD after 24 h that was less than what was observed for the 4-kan cultures (compare Fig. 1A and C, and Supplementary Fig. S3B).

We studied the kanamycin resistance of the bacterial population grown after P0 in 4-kan or 8-kan. For the 4-kan populations, MIC assays were performed on a sample drawn at 24 h (Fig. 1B). For the 8-kan cells, these assays were performed on individual isolates derived from the 24-h-old P0 culture (Fig. 1D). This difference in approach was adopted, because replicate 8-kan cultures grown in flasks for these experiments appeared to show more variable growth characteristics than the 4-kan cultures (Fig. 1A and C); note however that this cannot be statistically supported by data from a larger number of cultures grown in plates (Supplementary Fig. S3). The 4-kan populations displayed an increase in MIC of ∼2-fold compared with the initial inoculum (Fig. 1B, red bars). Among the clones derived from the 8-kan populations, a few were only slightly different from the *wild type*, whereas others displayed an increase of ∼2- to 5-fold (Fig. 1D, P0 8-kan). As a control, bacteria grown in plain LB showed no increase in kanamycin resistance (Fig. 1B—black bars; for *wild type* control colonies refer to the right side panels in Fig. 1D).

After the first 24 h culture, the bacteria were diluted into fresh medium containing the same concentration of the antibiotic (i.e. P1). The MIC of the 4-kan population was determined again after entry into stationary phase and found to be ∼4-fold higher than a control population that had not been exposed to the antibiotic (Fig. 1B, red bars). Further serial passages in 4-kan did not lead to substantial changes in the MIC of the population, suggesting that a stable variant had emerged. In the 8-kan populations, after dilution into fresh medium, most resultant isolates displayed >4- to 5-fold increase in MIC over the *wild type* (Fig. 1D, 8-kan P1 colonies). We do not attempt a direct, time-anchored comparison of MICs between the 4-kan and 8-kan cultures here, since the experiment for the 8-kan populations included a plating step to isolate colonies, which was not performed for the 4-kan cultures (see Supplementary Fig. S4 for time-anchored population MICs for a separate set of experiments).

**Figure 2.**
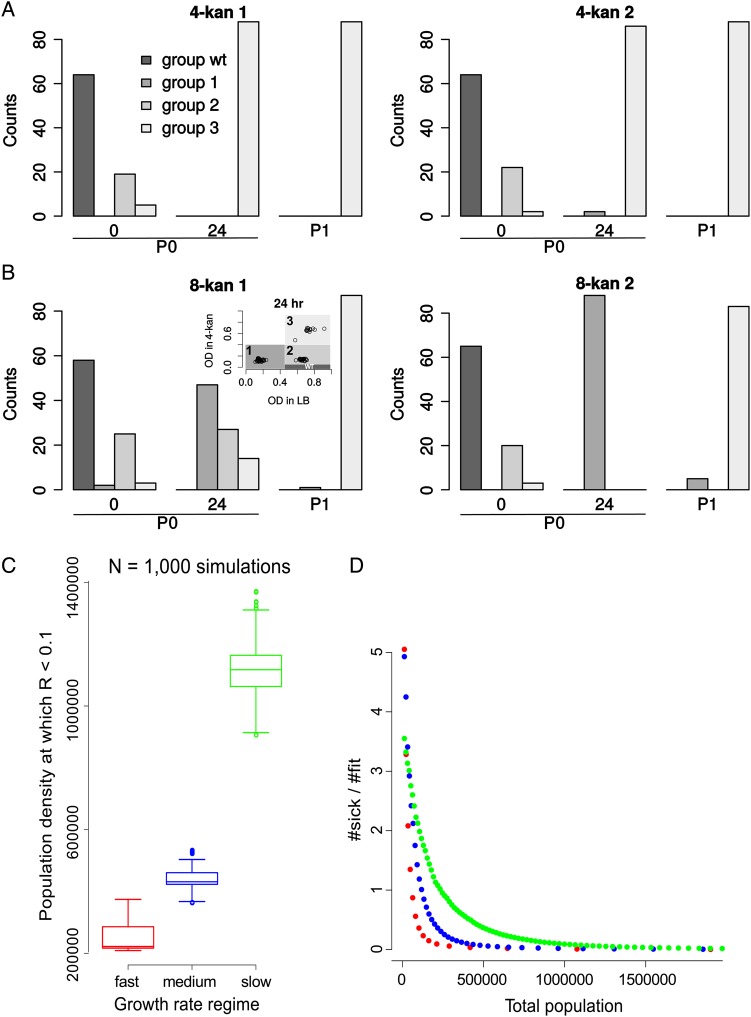
Population structure of evolving populations. (A) *E. coli* populations grown in 4-kan are more homogeneous in composition and comprised mostly of fit variants at 24 h. Isolates were classified into several groups based on the stationary-phase OD_600_ reached in 4-kan (tolerance to kanamycin) and 0-kan (growth in the absence of kanamycin; see inset in B). Plotted are counts of different types of isolates. *Wild-type-*like isolates (black bars) have been arbitrarily defined for the analysis as non-tolerant but fit isolates (falling in the OD range indicated in the inset of B) and predominate at the start of the experiment (0 h.). Group 1 (dark grey) isolates are tolerant but sick variants. Group 2 (grey) and 3 (light grey) are tolerant and fit variants (see inset of Figure 1B for definition of groups based on stationary-phase OD_600_ in plain LB and 4-kan and Supplementary Figure S5 for all plots related to this figure as well as plots for 0-kan control populations in which the *wild-type*-like colonies predominate throughout the course of the experiment). The counts change with time during the course of the batch evolution experiment (0 h, 24 h and P1). Results of the first replicate are displayed in the left panel and the second replicate in the right panel. (B) Populations grown in 8-kan are composed of multiple subpopulations, with the sick variants dominating at 24 h. Thus, tolerant but sick variants predominate in the first batch growth in 8-kan, and subsequent passaging results in an increase in number of the fit variants. (C) 1,000 independent growth simulations were performed for three growth regimes. This plot shows the distributions of the total population size (a reflection of the number of iterations or time) at which the ratio of the sick mutant to the fit mutant falls below 10%. Distributions are represented by box plots. The box represents the inter-quartile range and the line within the box indicates the median value. The maximum and the minimum value (1.5 times the inter-quartile range), excluding the outliers, is represented by the whiskers. The points represent the outliers. Probabilities of division per iteration are as follows. For the slow growth regime (green), µ_wt_ = 0.01, µ_sick_ = 0.03 and µ_fit_ = 0.1; for the intermediate growth regime (blue), µ_wt_ = 0.03, µ_sick_ = 0.1 and µ_fit_ = 0.3; for the fast growth regime (red), µ_wt_ = 0.1, µ_sick_ = 0.3 and µ_fit_ = 1. (D) A scatter plot of the ratio of the number of sick to fit mutants as a function of the population size (equivalent to the number of iterations or time) for an example simulation from (C).

What are the characteristics of the bacterial population resulting from the above-described growth in the antibiotic, in terms of its ability to grow in the presence or absence of kanamycin? For this, we first grew cells—produced by P0 cultures in antibiotic-free, 4-kan or 8-kan media—on LB agar plates. Individual colonies so obtained were then checked for their ability to grow in the absence of kanamycin (plain LB, 0-kan), moderately sublethal levels of kanamycin (4-kan) and lethal levels of kanamycin (20-kan). We note here that this experimental strategy is limited in the sense that the growth experiments performed to assess fitness also contribute to further evolution and selection. However, these steps were performed in the same manner for 4-kan and 8-kan populations, and therefore, the results thus obtained enable a direct comparison between the two antibiotic concentrations. Control colonies derived from all the populations immediately after inoculation displayed *wild-type* characteristics: good growth in 0-kan, poor growth at 4-kan and no growth at 20-kan (Supplementary Fig. S5A–F, 0 h.). Similarly, colonies derived from populations not treated with kanamycin retained this response throughout the course of the experiment (Supplementary Fig. S5A and S5D). Most colonies derived from 4-kan populations grew well in LB and in 4-kan; in addition, they were more resistant to 20-kan than the *wild type* (Fig. 2A, and Supplementary Fig. S5B and S5E). In contrast, a proportion of colonies derived from 8-kan populations, though more resistant to the antibiotic than the *wild type*, showed severe growth defects (Fig. 2B, and Supplementary Fig. S5C and S5F). The proportion of colonies with a growth defect varied between trials (Fig. 2B, compare group-1 and -2 colonies in 8-kan 1 and 2 at 24 h, between the two replicates). After another passage in the same concentration of the antibiotic (P1), both 4-kan and 8-kan cultures produced antibiotic-resistant colonies with little growth defect (Fig. 2A and 2B, group-3 colonies increase at P1).

In summary, growth in 4-kan quickly results in a population of bacteria that are not only resistant to the antibiotic, but also display little defect in growth in the absence of the antibiotic. On the other hand, many sick but resistant mutants persist longer during growth in 8-kan. We suspect that the difference between the two antibiotic regimes in the trade-off between growth and antibiotic resistance might explain our observations. The lower toxicity, at lower antibiotic concentration, leads to higher growth rates. This could select for variants with low fitness defects; resistant, but sick bacteria might be competed out rapidly. On the other hand, the greater lethality imposed by the higher antibiotic concentration allows the persistence of sick bacteria for a longer time. To test this further, we performed *in silico* stochastic growth simulations in which two types of antibiotic-resistant mutant bacteria—one with high growth and the other with low growth—were competed against each other and with a very slow-growing, antibiotic-sensitive *wild type* (see Methods and Supplementary Fig. S6). These simulations suggest that the slow-growing mutant will be out-competed more rapidly in an environment supporting higher growth rates in general (e.g. 4-kan) than one where overall growth rates are low (8-kan) (Fig. 2C and 2D). Our analysis of colonies had also suggested tentatively that the manner in which stationary-phase OD varies with the concentration of the antibiotic might differ across populations: while many of the fast-growing colonies show a sharp decline in growth from 4-kan to 20-kan, the slow-growing variants typically show less drastic decline in growth in 20-kan compared with 4-kan (Supplementary Fig. S7). This will only accentuate the trends observed in our simulations.

### 3.2. Representative low-cost mutations are in the translation elongation factor EF-G

**Figure 3.**
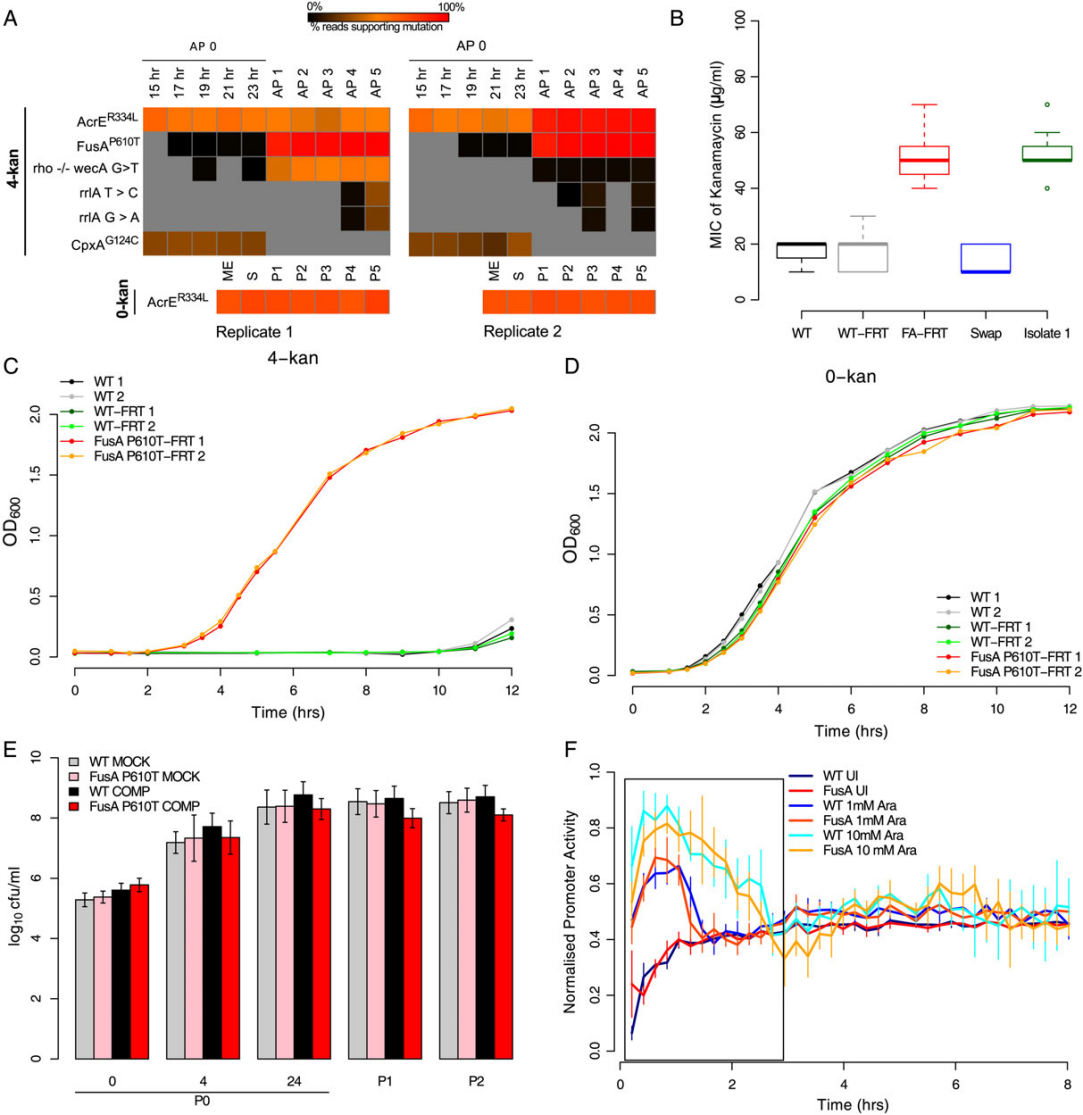
FusA^P610T^ confers low-cost resistance to kanamycin. (A) Heat maps indicating the proportion of reads supporting a given mutation (red—100% of reads support the mutation; black—close to 0%, i.e. very low; grey—0%, i.e. not detectable). Each column represents a time point, and the mutations are represented by rows (AP0-5: passages in 4-kan; 15–23 h: time points in AP0; ME, S and P1-5: mid-exponential- and stationary-phase time points in passage 0 and passages 1 through 5 in 0-kan; replicates are indicated below the heat map). Standard single letter amino acid representations have been used to denote substituted reside at the indicated position in superscripts. Intergenic region (IGR) mutations are represented by a ‘−/−’ between the genes flanking the IGR and the nucleotides found modified are indicated with a ‘>’. For simplicity, mutations found only in 4-kan populations are shown. However, since AcrE^R334L^ was also present in the control, the mutation is included separately below. While the mutation in *fusA* reaches (true, i.e. 100%) fixation in 4-kan populations by P3, it is also detected in the initial round of growth (P0) albeit at very low levels. AcrE^R334L^ fixes along with FusA^P610T^ in the second replicate but remains in the first replicate at an intermediate level. This maintenance can be explained only if FusA^P610T^ arose again in this background (see Supplementary Figure S8: Sanger-sequenced isolates suggest this scenario). A mutation in *cpxA* is also seen in AP0 in both replicates, but it is lost in subsequent passages as the mutation in *fusA* picks up. (B) The FusA^P610T^ mutation alone confers ∼3-fold resistance to kanamycin. This panel shows box plots (as defined in description of Figure 2C) of MICs (*n* = 8) for the *wild-type* strain into which the FusA^P610T^ mutation was transferred by phage transduction (FusA^P610T^-FRT; named so as the FRT site remains near the gene after the process). Controls *wild type* (WT), *wild type* with the FRT site at the same locus (WT-FRT) and the (back-swap) control in which the FusA^P610T^ mutation was replaced with the *wild-type* allele by phage transduction are also shown along with a representative 4-kan population isolate in which the FusA^P610T^ mutation was confirmed by Sanger sequencing. (C) Growth curves of *wild type* (WT) and FusA^P610T^-FRT in plain LB (0-kan) and (D) in LB + 4 µg/ml kanamycin (4-kan). In the absence of kanamycin, the FusA^P610T^ strain grows almost as well as *wild type*. (E) The FusA^P610T^-FRT strain competes well against the WT-FRT strain in plain LB. Bold colours represent the competition experiment (see Methods), whereas the light colours represent the mock competition experiment (both strains were grown separately and mixed prior to plating). Plotted are log_10_ colony forming units (cfu)/ml versus the time point {error bars represent relative error in *y*, [0.434 * (d*y*/*y*)], from pooled multiple counts from two independent replicates; d*y*: standard deviation}. (F) Promoter activities (normalized to fall between 0 and 1) for GFP^mut2^ induction are similar in the *wild type* and the FusA^P610T^ mutant, suggesting the absence of any defects in translation in this mutant. GFP^mut2^ induction from the P_BAD_ promoter in WT-FRT and FusA^P610T^-FRT for two different concentrations of arabinose is shown (1 mM: 1 Ara; 10 mM: 10 Ara and uninduced control: UI). Induction was done in plain LB; period of peak promoter activity is represented by a box (error bars indicate standard deviation for four replicates). For induction in presence of kanamycin, see Supplementary Figure S10C.

To characterize the mutational trajectories taken by *E. coli* towards increased resistance in 4-kan, we performed deep sequencing of the (meta-)genomes of the bacterial populations at various time points during growth in the antibiotic. Two biological replicate cultures were analysed. We performed gDNA sequencing at high temporal resolution for the P0 culture (Fig. 3A). As mentioned earlier, over the course of this culture, the MIC increased ∼2-fold from the basal level. As a control, growth was monitored in an antibiotic-free medium and gDNA sequenced from mid-exponential- and stationary-phase populations. For subsequent dilutions into antibiotic-containing media (P1, P2, etc.), sequencing was performed after entry into stationary phase.

To catalogue the constituting members of the heterogeneous population resulting from growth of *E. coli* in 8-kan, we first plated 24-h-old 8-kan populations (P0 and P1) on LB agar plates. We selected multiple isolates, representing the range of MIC and growth rates observed, from four biological replicates for gDNA sequencing (Fig. 1D). From each of P0 and P1, we sequenced 11 isolates, alongside one isolate from a control population grown in plain LB.

The only mutation that had gone to fixation in the two replicate 4-kan cultures was one in *fusA* (FusA^P610T^), the gene encoding the translation elongation factor EF-G, which was found in the population obtained after P1 in 4-kan. The P0 culture in 4-kan had produced a genetically heterogeneous population of variants, with none supported by >31% of reads (e.g. AcrE^R334L^ mutation, also seen in *E. coli* not exposed to the antibiotic and probably not associated with kanamycin resistance as shown in Supplementary Fig. S2B, and CpxA^G124C^ in a sensory histidine kinase with known links to antibiotic resistance^49^; Fig. 3A, Supplementary Table S1). All these mutations were supported by high-quality Illumina sequence reads and gave no reason for suspecting artefactual data: no enrichment for being seen in sequences mapping to one strand and not particularly located at the ends of reads. Here we also note that we do not see any evidence for ribosomal gene amplifications (Supplementary Table S2). In contrast, at the end of P1, FusA^P610T^ was supported by >90% of sequencing reads. In retrospect, we noticed that the FusA^P610T^mutation was present at low levels (∼1%) in sequencing reads from the P0 culture in 4-kan, showing that it had started to develop during the earliest stages of the experiment, going to fixation at some point in P1. This mutation was commonly present alongside that in *acrE* and less frequently with a mutation in the intergenic region between *rho* and *wecA*, as determined by Sanger sequencing of PCR amplicons from single colonies (Supplementary Fig. S8A). The population (Fig. 1B), and various clones isolated from it (Supplementary Fig. S8B), showed ∼4-fold higher MIC for kanamycin, with no difference between clones carrying only the FusA^P610T^ mutation and those carrying the *rho*-*wecA* mutation alongside (Supplementary Fig. S8B and S8C). The FusA^P610T^ mutation was stable: it was found in genome sequences obtained after 5 passages (Fig. 3A), and we did not observe any change in MIC even after 10 serial passages in an antibiotic-free medium (Fig. 1B, green bars).

**Figure 4.**
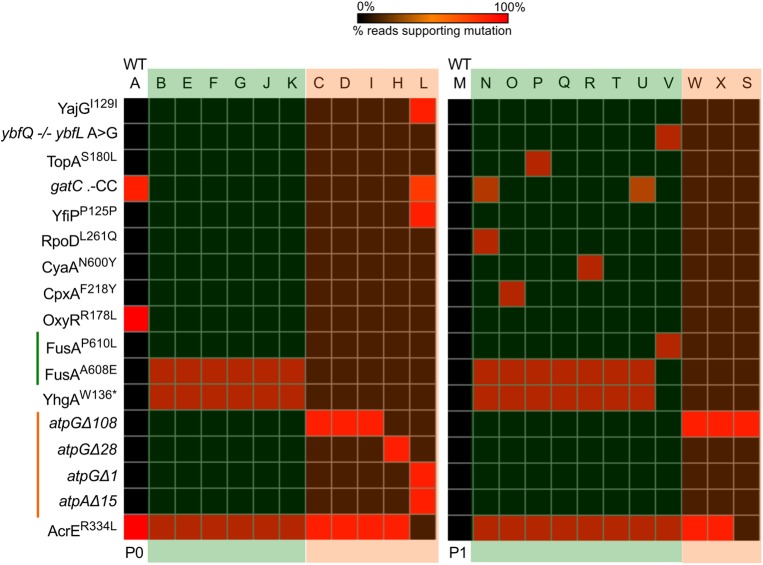
Deep sequencing of isolates reveal two groups of mutations in the 8-kan evolving populations. Heat maps with the presence/absence of mutations in the isolates. Each column represents an isolate, and the mutations are represented by rows. Passage number is indicated below. The control isolate is the first column in either passages (indicated by WT). Both 0%, i.e. not detectable, and values close to 0% are represented with black for ease of visualization; for isolates, any low percentage SNPs can be ignored. The isolates can be easily put into two groups: those with a point mutation in *fusA* and those with deletion(s) in subunits of the ATP synthase complex. In P1, a constellation of other mutations can be seen. These second site mutations occur only in the FusA^A608E^ background. Also, another *fusA* variant (FusA^P610L^) emerges in this passage. Standard single letter amino acid representations have been used to denote substituted residue at the indicated position in superscripts. ‘*’ indicates a STOP mutation. Δ indicates a deletion of nucleotides indicated by the number beside the symbol. Intergenic region (IGR) mutations are represented by a ‘−/−’ between the genes flanking the IGR and the nucleotides found modified are indicated with a ‘>’. Insertion of ‘two cytosines’ in the gene *gatC* is represented as .-CC.

We identified two other FusA mutations in the 8-kan cultures, in six (out of 11) colonies from the P0 culture and eight (out of 11) colonies from P1 (Fig. 4, Supplementary Table S3). The most common mutation was FusA^A608E^, located close to the FusA^P610T^ mutation found in our 4-kan cultures. The FusA^A608E^ mutation was found alongside a stop mutation in *yhgA* (YhgA^W136*^); given the obvious importance of *fusA* mutations to kanamycin resistance, it is likely that the *yhgA* mutation is not contributing and is probably a hitch-hiking candidate. All the FusA^A608E^-containing clones had a mutation in *acrE* (AcrE^R334L^), which was also present in the no-antibiotic control isolate sequenced. Yet another EF-G mutation was found in a single colony obtained from P1: this mutation affected the same residue as that in the 4-kan cultures, with P610 mutated to a leucine (FusA^P610L^) instead of a threonine seen in the 4-kan population.

**Figure 5.**
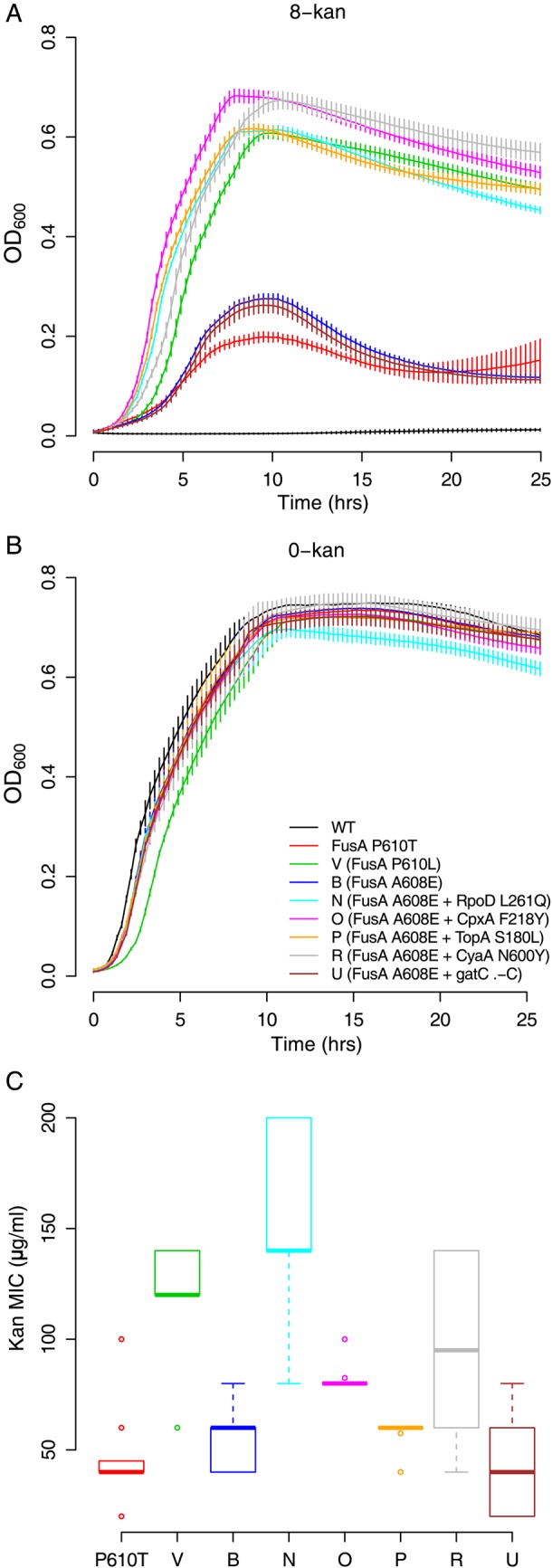
Multiple EF-G mutations confer kanamycin resistance which is increased by second site mutations. (A) Growth curves of the EF-G mutants in 8-kan and (B) plain LB (error bars represent standard deviation from eight replicates; legend from B is applicable throughout the figure). The stationary-phase OD_600_ for the isolates containing the FusA^A608E^ alone, and in combination with *gatC* .-CC (cytosine insertions) mutation, is lower in 8-kan than for any of the other mutants. This suggests that the second site mutations in RpoD, CpxA, TopA and CyaA may improve FusA^A608E^ strain's ability to grow at low kanamycin concentrations. Of note, the FusA^P610T^ mutation isolated from 4-kan populations does not grow as well as the other *fusA* mutant strains in 8-kan. (C) Kanamycin MICs (*n* = 8; box plots as defined in description of Figure 2C) for the isolates with the mutations in EF-G (*fusA*). The FusA^P610L^ mutation might provide higher resistance to kanamycin than the FusA^P610T^ mutation (*P* < 10^−5^; two-sample independent *t*-test). However, the FusA^A608E^ mutation alone or in combination with *gatC* .-CC (cytosine insertions) or TopA^S180L^ confers resistance levels similar to the FusA^P610T^ mutation (*P* = 0.289, 0.817 and 0.151, respectively). In combination with CyaA^N600Y^, CpxA^F218Y^,and RpoD^L261Q^, the FusA^A608E^ mutation does better and has higher resistance than either FusA^P610T^ or FusA^A608E^ alone (*P* = 0.006, 0.0003 and <10^−5^, respectively). The emergence of some of these mutations in P1, although a resistant mutation is already present, may be explained by this observation.

The FusA^P610L^ mutation grew almost as well in 8-kan (Fig. 5A) as did the *wild type* in 0-kan (Fig. 5B) and FusA^P610T^ in 4-kan (Fig. 3C). However, the FusA^P610T^ mutation performed relatively poorly in 8-kan (Fig. 5A), suggesting that its effect is more restricted to lower kanamycin concentrations. The difference between the two mutations at the same site may also be reflected in the higher MIC of the FusA^P610L^ variant (Fig. 5C). This isolate had also an intergenic mutation at another locus (*ybfQ* -/- *ybfL*); at this point, we do not know whether this adds to the resistance phenotype of FusA^P610L^. We also noticed that FusA^P610L^ grew much better than FusA^A608E^ in 8-kan (Fig. 5A), despite the two being obtained from the same culture conditions and carrying mutations in adjacent residues. Consistent with this observation, FusA^A608E^-containing isolates were embellished with additional mutations in P1, but not in P0 (Fig. 4—P1). These, admittedly curious, mutations include RpoD^L261Q^, CpxA^F218Y^, TopA^S180L^ and CyaA^N600Y^. It is notable that the FusA^A608E^ + CpxA^F218Y^/TopA^S180L^/CyaA^N600Y^/RpoD^L261Q^ double mutants showed higher growth than the FusA^A608E^ single mutation alone in 8-kan (Fig. 5A), though the single and the double mutations did not differ from each other in growth in the absence of the antibiotic (Fig. 5B). And the MIC of the double mutants was higher when FusA^A608E^ was present in combination with RpoD^L261Q^ and CyaA^N600Y^; with the others, the increase was not so evident (Fig. 5C). These might suggest genotype-dependent fine tuning of adaptation to the selected concentration of the antibiotic.

The above analysis show that several mutations in *fusA* arise in cultures grown in kanamycin and spread through the population rapidly. All the three mutations are present at the carboxy-end of Domain 4 of EF-G, the tip of which interacts with the decoding site of the ribosome (Supplementary Fig. S9). To specifically investigate the contribution of FusA^P610T^ to kanamycin resistance and its cost in terms of growth in the absence of the antibiotic, we used phage transduction to move this mutation into a parental *wild-type* strain. This strain now had the FusA^P610T^ mutation in addition to an FRT site downstream of *fusA* (between the *rpsG*-*fusA*-*tufA* transcription unit and *chiA*). Genome sequencing of this clone showed that no other mutation had been introduced during transduction (data not shown). This strain is henceforth referred to as the *fusA^mut^::FRT* strain. The characteristics of *fusA^mut^::FRT* strain were compared with a *wild type* carrying the FRT site in the same region (*wt::FRT*) and with the parental *wild type*. We measured the MIC of the *fusA^mut^::FRT* strain and found it to be comparable with those of the clones carrying the mutation (Fig. 3B, compare FA-FRT and Isolate 1). The *wt::FRT* showed no resistance, similar to the *wild type* without the FRT site. The *wt::FRT*, as well as the parental *wild type*, showed little or slow growth in solid or liquid media containing 4-kan (Fig. 3C). However, the *fusA^mut^::FRT* strain grew as well in 4-kan (Fig. 3C) as the *wt::FRT* and the parental *wild type* in non-antibiotic medium (Fig. 3D). Finally, the *fusA^mut^::FRT* strain, when replaced with the *wt::FRT* allele by phage transduction, lost its resistance to kanamycin (Fig. 3B). These show that the FusA^P610T^ mutation contributes almost entirely to the resistance of the 4-kan evolved population.

The following set of growth experiments allowed us to confirm that the FusA^P610T^ mutation does not result in a perceivable growth defect in the absence of the antibiotic: *fusA^mut^::FRT* could grow almost as well as *wt::FRT* in LB without kanamycin (Fig. 3D); the *fusA^mut^::FRT* strain grew well and conferred kanamycin resistance even at 42°C (Supplementary Fig. S10A), showing the absence of a temperature-sensitive phenotype in contrast to previously isolated FusA mutants^32^; the *fusA^mut^::FRT* strain competed well with *wt::FRT* strain in competition assays (Fig. 3E and Supplementary Fig. S10B for competitions with *wild type* without the FRT site). Finally, the *fusA^mut^::FRT* strain did not seem to have defects in translation as seen from kinetics of induction of GFP^mut2^ from a tightly controlled arabinose-inducible promoter (pBAD18::*gfpmut2*) (Fig. 3F). For induction in the presence of kanamycin, see Supplementary Fig. S10C and D.

Hou *et al.*,^32^ in their report of a screen for temperature-sensitive mutations in EF-G, document two mutations: *fusA100* (FusA^G502D^) and *fusA101* (FusA^Q495P^). The screen was performed by selection of resistant colonies at 30°C on LB agar containing lethal concentrations of kanamycin. These two strains seemed to incur a heavy cost manifested by growth defects even at non-restrictive temperatures and had defects in translation as seen from kinetics of induction of a reporter gene (β-gal in their assay). In contrast, our study, by performing selection experiments at sublethal kanamycin concentration, has identified a group of mutations with little detectable defect in growth or induction of translation.

### 3.3. Mutations in ATP synthase confer costly, basal level resistance to kanamycin

Whole genome sequencing indicated that isolates from the 8-kan population (5 from P0 and 3 from P1), which did not contain a mutation in EF-G, contained deletions in genes encoding subunits of ATP synthase (Fig. 4). The deletions in the genes encoding subunits of ATP synthase were of different lengths: deletions of 108 bp (three isolates), 28 bp (1 isolate) and 1 bp (1 isolate) were seen in *atpG*, encoding the γ-subunit of the ATP synthase complex; and a deletion of 15 bp (1 isolate) was seen in *atpA* encoding the α-subunit. The isolate with the 15 bp deletion in *atpA* also had the 1 bp deletion in *atpG*. A recent large-scale study of aminoglycoside resistance mutations had identified mutations not only in the ATP synthase, but also in other members of the oxidative phosphorylation pathway.^33^ This suggests that inactivation of the ATP synthase complex and oxidative phosphorylation must be a general mechanism towards resisting kanamycin. Whereas the three types of ATP synthase mutations described above were found in P0, only the 108 bp deletion in *atpG* could be recovered in P1 (Fig. 4).

**Figure 6.**
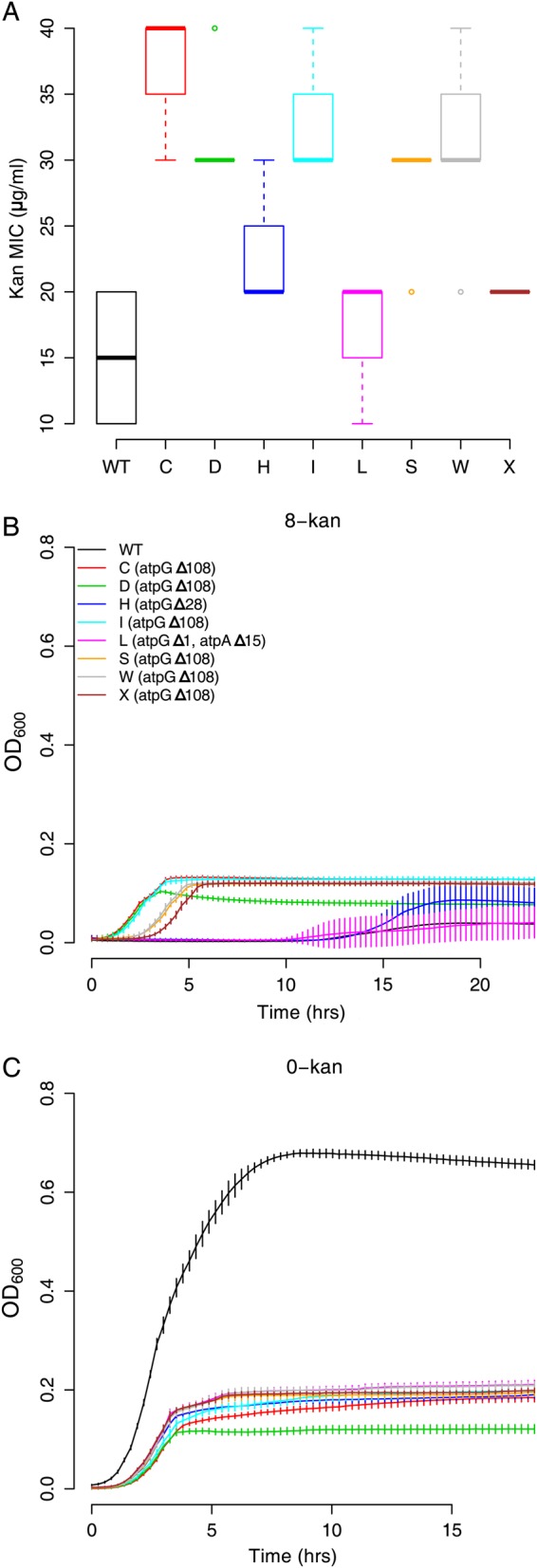
Deletions in subunits of the ATP synthase gene are costly mutations that confer kanamycin resistance. (A) Kanamycin MICs (*n* = 8, box plots as defined in description of Figure 2C) for the isolates with the deletions (two-sample independent *t*-tests for *wild type* against isolates yield *P* values < 10^−5^ for all except isolates L: *P* = 0.0095 and X: *P* = 0.000879). Thus, these deletions increase resistance to kanamycin only slightly. (B) Growth curves of the ATP synthase mutants in 8-kan and (C) 0-kan (error bars represent standard deviation from eight replicates; legend from B is applicable throughout the figure). In plain LB, the ATP synthase mutants grow to a much lesser extent than *wild type*, suggesting a huge cost factor associated with these deletion mutations. The strains with the 108 bp deletion in *atpG* are able to grow in 8-kan to approximately the same extent as in the absence of kanamycin. Oddly though, isolates H (*atpG*Δ28) and L (*atpG*Δ1, *atpA*Δ15), although possessing a similar mutation (refer Supplementary Figure S11), do not grow well in 8-kan. This is consistent with these strains not being picked up in the P1 (Fig. 4), although limited sampling could also result in this.

We analysed the isolates in which the ATP synthase mutations were present in further detail. All the three mutations in *atpG* are expected to *delete* the C-terminal end of the γ-subunit of the F0 component of the ATP synthase (Supplementary Fig. S11). Since most of these strains are fully sequenced and do not possess any other mutations apart from the AcrE^R334L^ that does not confer kanamycin resistance, we could work directly with these strains. Surprisingly, only the isolates with the 108 bp deletion in *atpG* seem to be around 1.5- to 2-fold resistant to kanamycin (Fig. 6A) and grow well in 8-kan (Fig. 6B), while the other deletion strains do not. Regardless of the resistance phenotype, all of these strains have incurred a severe cost as a result of the deletions as seen from their growth in plain LB (Fig. 6C). Consistent with this, there is a relative decline in the abundance of ATP synthase mutants in P1 compared with P0, with a concomitant increase in the abundance of the less costly mutations in FusA.

Taken together, genome-sequencing experiments show that EF-G variants represent low-cost, kanamycin-resistant mutations. Several mutations, mapping to the same or adjacent residues in EF-G, confer resistance to the antibiotic. On the other hand, mutations in ATP synthase confer resistance to kanamycin, but also display severe growth defects.

## 4. Discussion

Antibiotic resistance is a major problem in health today. In particular, development of resistance under sub-inhibitory concentrations of an antibiotic is of interest. Bacteria are believed to be routinely exposed to sublethal concentrations of various antibiotics in their natural environments over long periods of time. In a clinical setting, improper adherence to a dose regime could lead to low levels of antibiotics in the body. Mutations conferring resistance to antibiotics target essential genes which may tolerate mutations poorly. Therefore, they represent a balance between the level of resistance they confer, as well as a possible loss of fitness—in the absence of the antibiotic. In addition, the accessibility of a resistance-conferring mutation also affects the kinetics of the evolution of resistance.

In this study, we observe that resistance of laboratory *E. coli* to two sublethal concentrations of the aminoglycoside kanamycin eventually produces a mutation that provides sufficient resistance to the antibiotic, while being of low cost as measured by growth or fitness in the absence of the antibiotic. This is only to be expected given enough time for selection to act as long as a less costly mutation (or a combination of mutations) for resistance is available to the bacterium. In a recent, intriguing report, an antibiotic resistance-conferring mutation was found in fact to enhance fitness of *Salmonella*.^50^

In cases where the levels of resistance conferred by a mutation are negatively correlated with its fitness, one would expect resistance to be persistently associated with a growth defect. Our experimental conditions presumably do not impose such a trade-off, though this needs to be systematically evaluated. We observe however that the selection under high sublethal concentrations of kanamycin results in the longer persistence of growth-defective variants in the population. This is consistent with the prior isolation of two defective, but resistant mutations by selection under lethal kanamycin concentrations.^32^

A representative low-cost mutation we have isolated is in the translation elongation factor EF-G. It is surprising that at least one of the mutations here displays little defect in growth or in inducing translation of GFP *in cellulo*. Further, the mutated residue P610, which we have investigated in some detail here, and A608 are both highly conserved as observed from multiple sequence alignments of EF-G from >2,000 bacterial genomes (Supplementary Fig. S12). Analysis of this residue in the context of EF-G structure shows that it is located at the C-terminal end of ‘Domain IV’ (DIV; Supplementary Fig. S9), whose tip interacts with the ribosomal decoding site. Mutation from a Proline to any other residue (Threonine and Leucine in our study; Glutamate in a recent screen for aminoglycoside resistance^33^) might increase the flexibility in movement of DIV, a feature that appears to play important roles during translocation.^51^

The levels of resistance conferred by the P610T mutation to a panel of aminoglycosides (Supplementary Fig. S13), in the context of a recent single-molecule study^15^ investigating the contribution of translocation inhibition to the activities of these antibiotics, suggest that its contribution to resistance is consistent with an effect on translocation. Aminoglycosides also affect ribosome recycling^16^; however, the mutation we describe is unable to restore a temperature-sensitive phenotype of a mutation in the ribosome release factor (Supplementary Fig. S14). In summary, a mutation at the C-terminal end of DIV of EF-G confers low-cost resistance to 2-DOS aminoglycoside antibiotics in a manner probably consistent with an effect on ribosome translocation. We note here that, as described in the ‘Results’, not all EF-G mutations—despite targeting the same site or adjacent sites—are equal.

At the other end of the fitness scale is the set of mutations we have identified in the ATP synthase. Most of these mutations are in the gamma component of the F0 subunit. The gamma polypeptide is the shaft that couples the proton gradient-driven rotation of the F1 subunit to the catalytic region on the F0 subunit

All the mutations in the gamma polypeptide, either as frame-shifts or as in-frame deletions, remove the C-terminal ∼40 residues of the polypeptide (Supplementary Fig. S11B). This end of the polypeptide cosily nestles in a region surrounded by the catalytic components of the ATP synthase (Supplementary Fig. S11C). It is thus reasonable to expect that these mutations abolish ATP synthase activity. Though *E. coli* should be able to synthesize ATP via substrate-level phosphorylation, oxidative phosphorylation culminating in ATP synthase activity is the major source of ATP. Thus, as expected, these mutations in ATP synthase result in severe growth defects. How inactivation of the ATP synthase can confer resistance to aminoglycosides is open to debate. To our knowledge, there are two relevant models in the literature. A recently published large-scale screen for aminoglycoside resistance identified members of the oxidative phosphorylation pathway as a major class of targets of mutation.^33^ These researchers report that a reduction in the PMF, as a consequence of these mutations, increases aminoglycoside resistance by decreasing the uptake of these antibiotics. On the flip side, these mutations increase sensitivity to other classes of antibiotics, which require PMF for efflux. A second model is related to the generation of reactive oxygen species. It has been suggested that many antibiotics kill their target cells by producing reactive oxygen.^52^ Inhibition of ATP synthase activity would eventually result in the cessation of oxidative phosphorylation, thus reducing reactive oxygen production. However, this model remains controversial and the jury is out to decide.^24,26^

Besides representing two extremes in the fitness landscape, the two mutations described here also represent two levels of ‘accessibility’. The space of mutations for ATP synthase inactivation is potentially large and, therefore, easily achieved. On the other hand, a mutation in EF-G, which specifically affects a certain function, resulting in low-cost aminoglycoside resistance is likely to be relatively rare. Despite this, the fact that the mutation in EF-G is more commonly observed in our experiments is a reflection of the considerable over-compensation of the difference in accessibility by that in fitness, both in the presence and in the absence of the antibiotic.

Mutations in EF-G may also indicate fine tuning of adaptation to narrow windows of antibiotic concentration: a mutation selected in a low sublethal concentration of the antibiotic resists a higher concentration poorly, though one selected in the latter performs better at this concentration despite the two mutations targeting the same amino acid residue (P to T versus P to L). In contrast, mutations in ATP synthase may confer a basal level of resistance that is robust to a broader range of antibiotic concentrations. This is suggested by a difference, between mutations in EF-G and those in ATP synthase, in the manner in which their growth decreases with increasing concentration of kanamycin. To visualize this, we first obtain 0-kan normalized MIC curves in which growth in (any) concentration of kanamycin, as measured by OD_600_ after 24 h, is normalized with respect to growth in the absence of kanamycin. Based on this, we find that the decline in growth with increasing antibiotic concentration is steeper for EF-G mutations than for ATP synthase mutations (Supplementary Fig. S15A).

In summary, our work points to the relevance of sublethal concentrations of an antibiotic to the rapid development of low-cost and resistant mutants, while also describing two classes of mutations—one obviously costly and the other less so—that confer resistance to a subset of aminoglycoside antibiotics.

## 5. Accession numbers

Genomic deep-sequencing data can be accessed from the NCBI Sequence Read Archive (accession number: SRP040449).

## Supplementary data

Supplementary Data are available at www.dnaresearch.oxfordjournals.org.

## Funding

The work was supported by the National Centre for Biological Sciences core funding. Funding to pay the Open Access publication charges for this article was provided by the National Centre for Biological Sciences, Tata Institute of Fundamental Research.

## Supplementary Material

Supplementary Data
