# Supplementary material for: Genomic Analysis Reveals Distinct Concentration-Dependent Evolutionary Trajectories for Antibiotic Resistance in Escherichia coli
Source: DNA Res. 2014 Oct 3;21(6):711–26. doi: 10.1093/dnares/dsu032 (PMC4263303; doi:10.1093/dnares/dsu032)
Supplement: Supplementary Data [file supp_21_6_711__index.html]

Genomic Analysis Reveals Distinct Concentration-Dependent Evolutionary Trajectories for Antibiotic Resistance in Escherichia coli — Supplementary Data 

# Genomic Analysis Reveals Distinct Concentration-Dependent Evolutionary Trajectories for Antibiotic Resistance in *Escherichia coli*

## Supplementary Data

Supplementary Data

**Files in this Data Supplement:**

- Supplementary Data - Pdf file
