# Supplementary material for: Genomic Analysis Reveals Distinct Concentration-Dependent Evolutionary Trajectories for Antibiotic Resistance in Escherichia coli
Source: DNA Res. 2014 Oct 3;21(6):711–26. doi: 10.1093/dnares/dsu032 (PMC4263303; doi:10.1093/dnares/dsu032)
Supplement: Supplementary Data [file supp_dsu032_dsu032supp.pdf]

Genomic Analysis Reveals Distinct Concentration-Dependent Evolutionary Trajectories for Antibiotic Resistance in Escherichia coli

Aalap Mogre, Titas Sengupta, Reshma TV, Preethi Ravi, Aswin Sai Narain Seshasayee

National Centre for Biological Sciences, Tata Institute of Fundamental Research, GKVK, Bellary Road, Bangalore, 560065, Karnataka, India

Supplementary Figure Details

Figure S1:

Overview of the evolution experiment.

The flowchart describes the evolution experiment carried out in two sublethal concentrations of kanamycin (4-kan: 4 µg/ml kanamycin, and 8-kan: 8 µg/ml kanamycin). P0, P1, etc. describe batch transfers of cultures every 24 hours into fresh medium as is depicted in the inset figure (lower left side). Light blue boxes indicate deep sequencing of gDNA extracted from entire evolving populations, whereas the darker blue boxes indicate deep sequencing of gDNA extracted from isolates obtained from the evolving populations. Green boxes indicate less costly mutants, whereas red boxes indicate costly mutants.

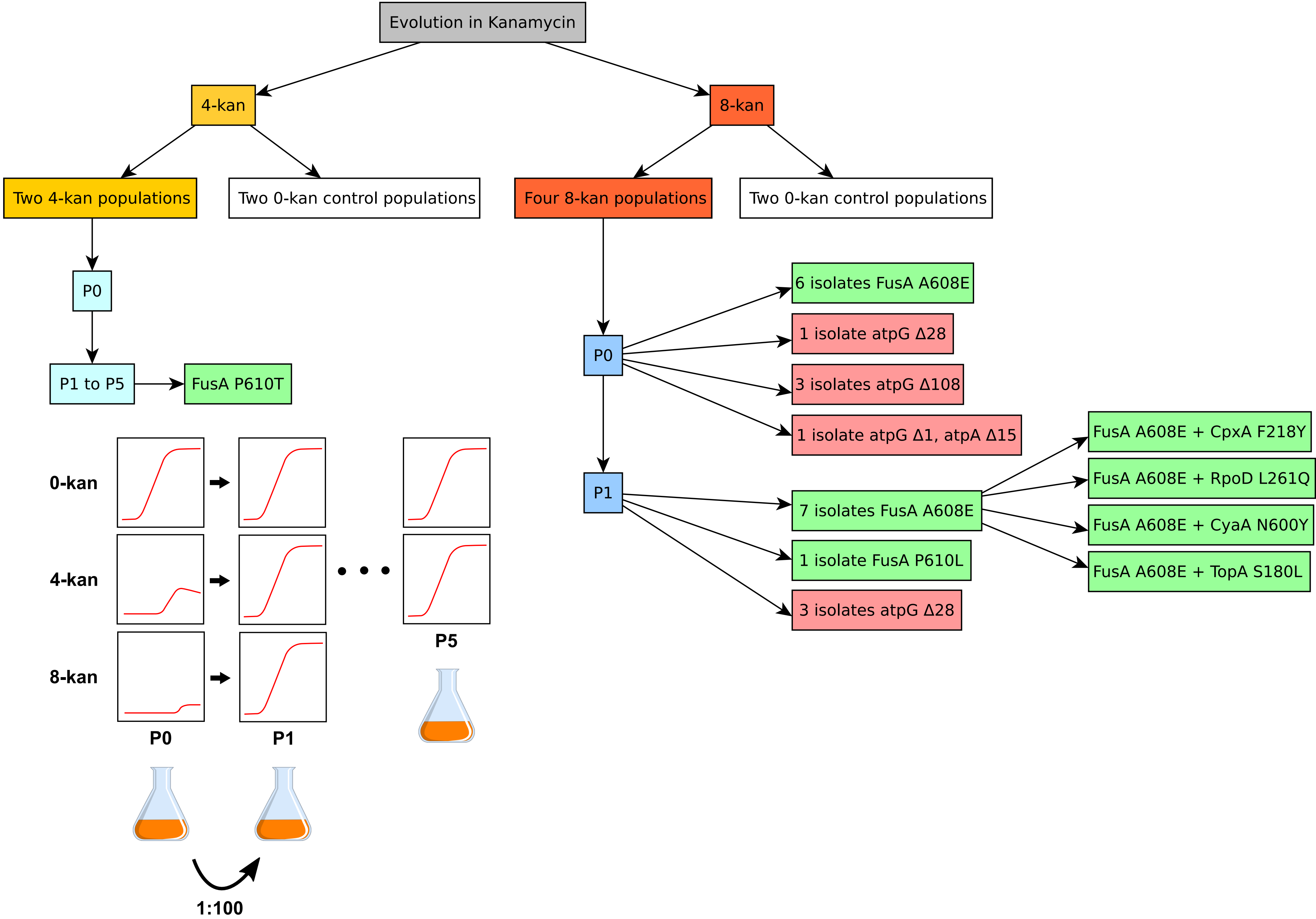

Evolution in Kanamycin
4-kan
8-kan
Two 4-kan populations
Two 0-kan control populations
Four 8-kan populations
Two 0-kan control populations
P0
P1 to P5
FusA P610T
P0
6 isolates FusA A608E
1 isolate atpG Δ28
3 isolates atpG Δ108
1 isolate atpG Δ1, atpA Δ15
P1
7 isolates FusA A608E
1 isolate FusA P610L
3 isolates atpG Δ28
FusA A608E + CpxA F218Y
FusA A608E + RpoD L261Q
FusA A608E + CyaA N600Y
FusA A608E + TopA S180L
0-kan
4-kan
8-kan
P0
P1
P5
1:100

Figure S2:

***Wildtype* MIC levels.**

(A) MIC curve of kanamycin for *wildtype E. coli* MG1655 with the sub-lethal range represented by the red gradient. The two concentrations ~25% and ~50% of the lethal level selected for the evolution experiments are indicated by the blue boxes (error bars indicate standard deviation derived from OD_{600} values of four technical replicates). MIC of *wildtype E. coli* is around 20 µg/ml (B) Since the $AcrE^{R334L}$ mutation was present in our *wildtype* populations, we determined the contribution of this mutation to kanamycin tolerance. Shown here are the kanamycin MICs for two isolates from our *wildtype* strain: one is *wildtype* for the gene ($AcrE^{WT}$) and the other contained the $AcrE^{R334L}$ mutation (n = 8). These are 'distribution' representations by box plots. The box represents the interquartile range and the line within the box indicates the median value. The maximum and the minimum value (1.5 times the interquartile range), excluding the outliers, is represented by the whiskers. The points represent the outliers. The $AcrE^{R334L}$ mutation does not confer tolerance to kanamycin.

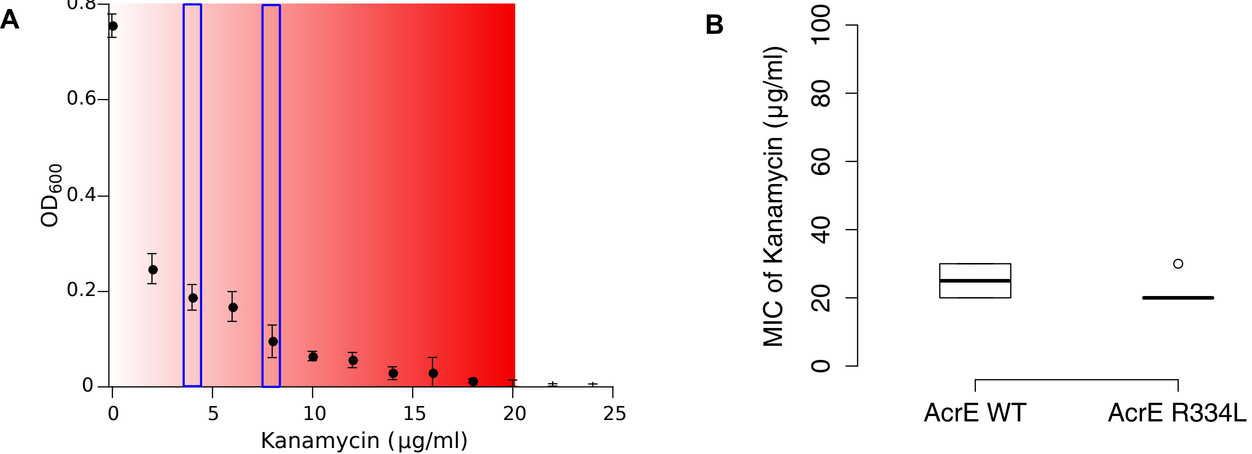
A
0.8
0.6
0.4
0.2
0
OD_{600}
0
5
10
15
20
25
Kanamycin (µg/ml)
B
MIC of Kanamycin (µg/ml)
100
80
60
40
20
0
AcrE WT
AcrE R334L

Figure S3:

Growth in 4 and 8-kan.

(A) Growth curves of *E. coli* MG1655 in 0-kan, 4-kan and 8-kan. These are results from around 94 replicates (0-kan) and 88 replicates (4 & 8-kan) of the parent population grown in a 96-well plate. (B) Growth parameters of the 4-kan and 8-kan populations: duration of lag phase, maximum growth rates and the stationary phase OD_{600}. Growth rates were calculated as d(lnOD)/ dt, and the maximal value was taken as the maximum growth rate. While growth rates and stationary phase OD_{600} are lower, the duration of lag phase is higher in the presence of kanamycin on average (p-values obtained from Welch's t-test, for any pairwise comparison is $< 10^{-16}$). These are 'distribution' representations by box plots. The box represents the interquartile range and the line within the box indicates the median value. The maximum and the minimum value (1.5 times the interquartile range), excluding the outliers, is represented by the whiskers. The points represent the outliers. Since these growth measurements were carried out in 96-well plates, growth parameters reported here will be different from those obtained from growth measurements in flasks.

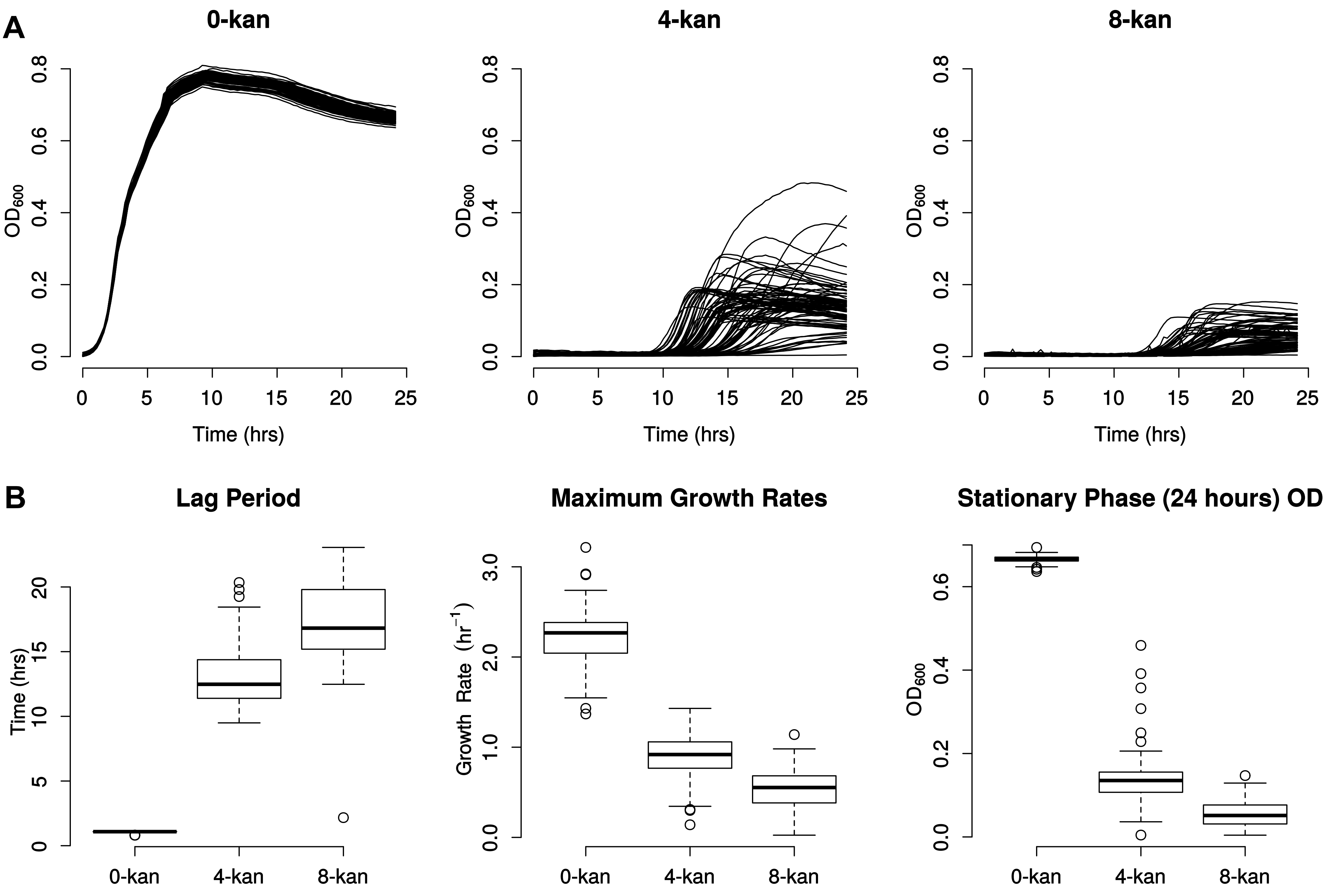
A
0-kan
4-kan
8-kan
OD600
Time (hrs)
B
Lag Period
Maximum Growth Rates
Stationary Phase (24 hours) OD
Time (hrs)
Growth Rate (hr−1)
OD600
0-kan
4-kan
8-kan

Figure S4:

MICs of evolving populations:

Population optical densities (OD_{600}) (A) and MICs (B) for an additional set of evolution experiments. 0,4 and 8-kan indicate that the cultures were grown in 0, 4 and 8 µg/ml of kanamycin. P0 and P1 indicate batch transfers of cultures. Different shades of grey represent different replicate populations evolved in parallel. Two control populations were maintained under 0-kan conditions, whereas four populations were maintained under 4 and 8-kan conditions in a 24 well plate (1ml).

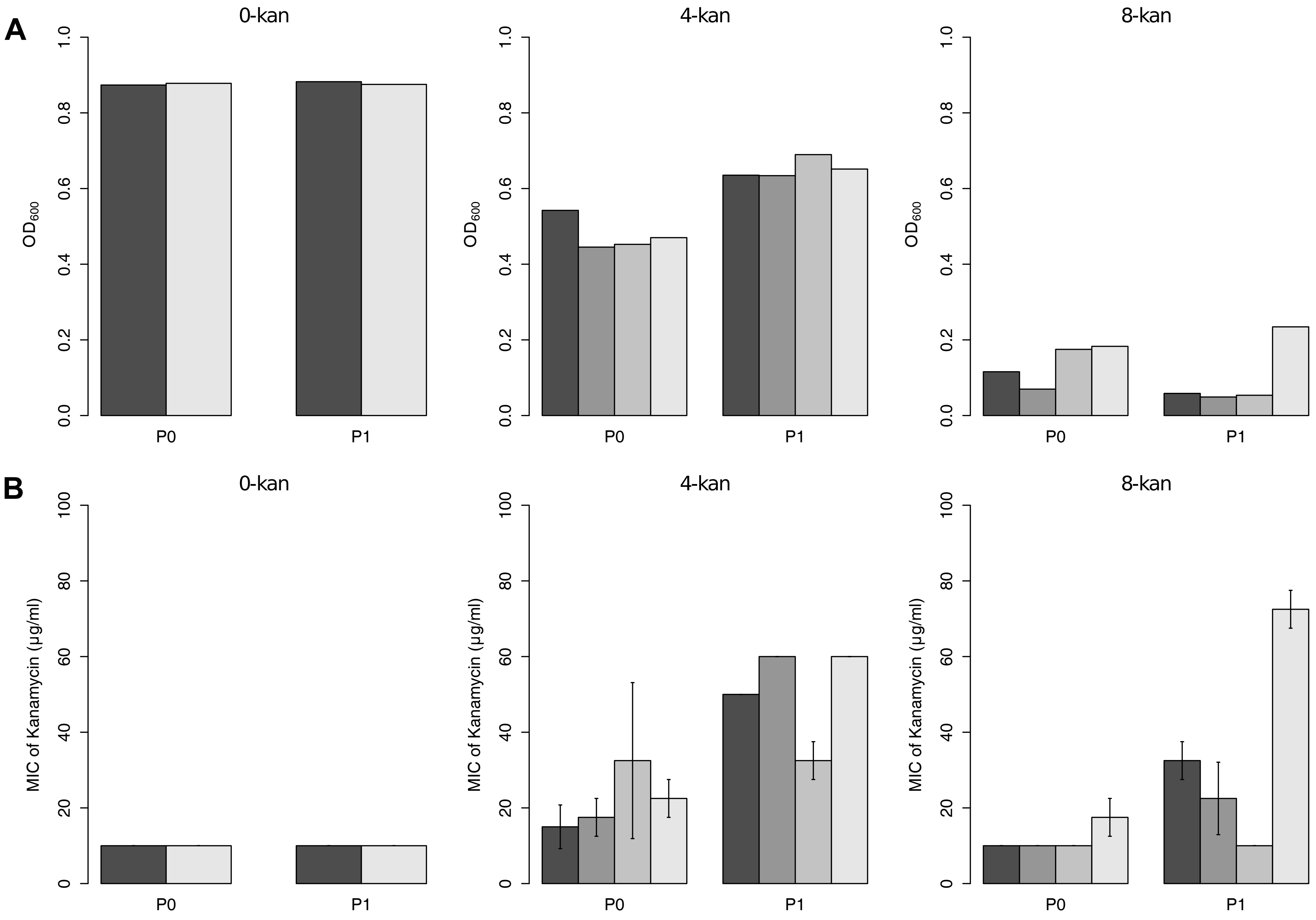

A
0-kan
4-kan
8-kan
OD600
P0
P1
B
MIC of Kanamycin (µg/ml)

Figure S5:

Population structure of evolving populations (part I).

(A-C) One trial, (D-F) another trial: growth characteristics of colonies derived from evolving and control populations, immediately after inoculation (0hr), in stationary phase (24hrs) and after the first passage (P1; indicated on the right) in medium with and without kanamycin (indicated on the top). Growth in plain LB is represented on the x-axis while growth in either 4-kan or 8-kan is represented on the Y-axis, both in terms of 24 hour OD_{600}. Blue box highlights the time-point where a difference in the evolving populations between the two different concentrations is evident. At this time-point the 8-kan populations are comprised of multiple sub-populations (sub-grouped based on their ability to tolerate kanamycin and their fitness in the absence of kanamycin). Note that the left panel in (C) 24 hours is identical to Figure 2B inset.

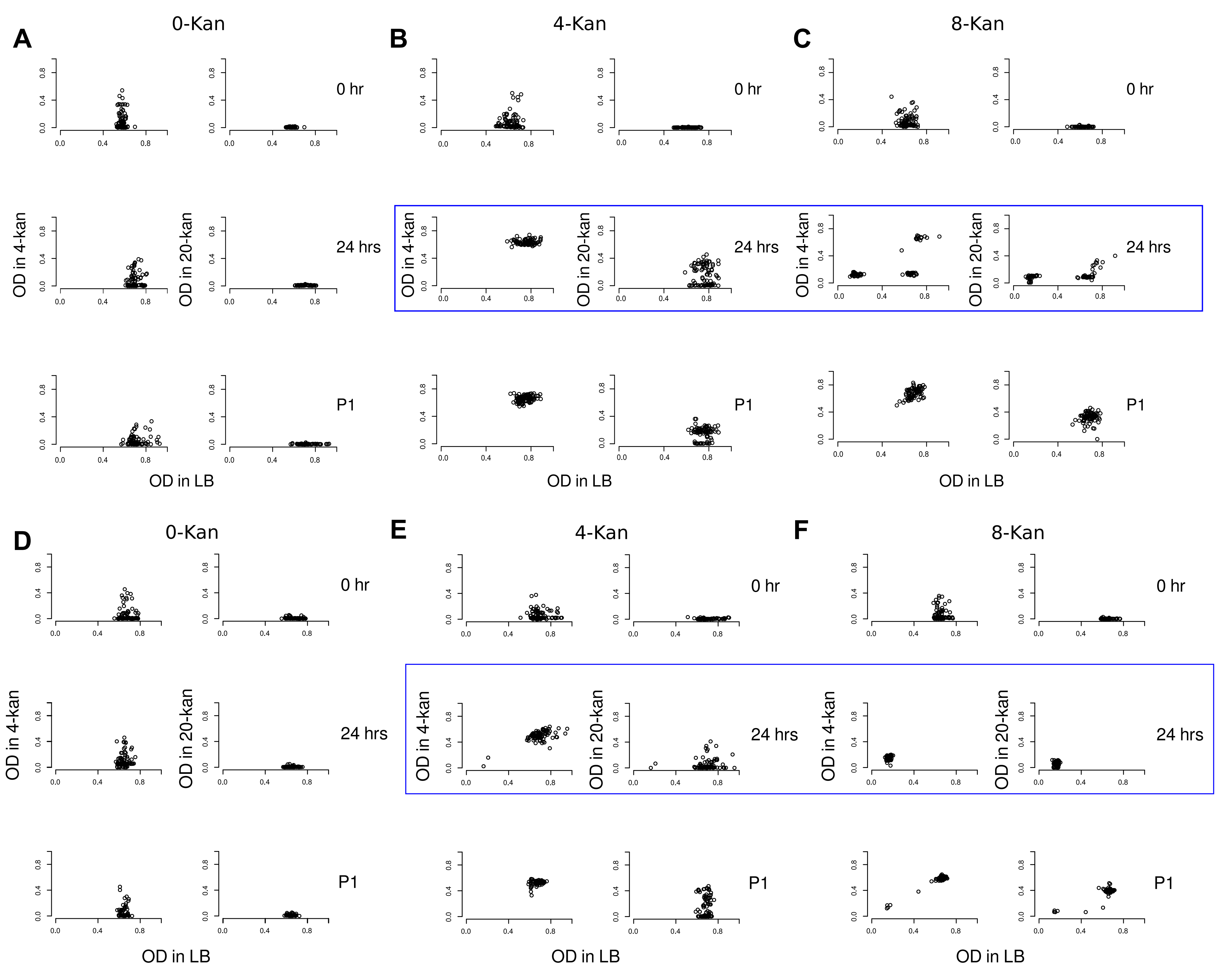

A
0-Kan
B
4-Kan
C
8-Kan
0 hr
24 hrs
P1
OD in 4-kan
OD in 20-kan
OD in LB
D
0-Kan
E
4-Kan
F
8-Kan
0 hr
24 hrs
P1
OD in 4-kan
OD in 20-kan
OD in LB

Figure S6

Schematic representation of growth simulation

The simulation, described in the Methods, involves tracking the sizes of three types of arrays (or hashes in this case, as the simulation code was written in PERL), each representing one type of bacterium. At each iteration, the sizes of the arrays increases depending in the probability of each member of the array doubling during that iteration. This set of probabilities are the primary parameters of the model. The initial size of the population was set to 10,000, with 1% of the cells being the fit mutants, 5% being the sick mutants and the remaining 94% being widtype.

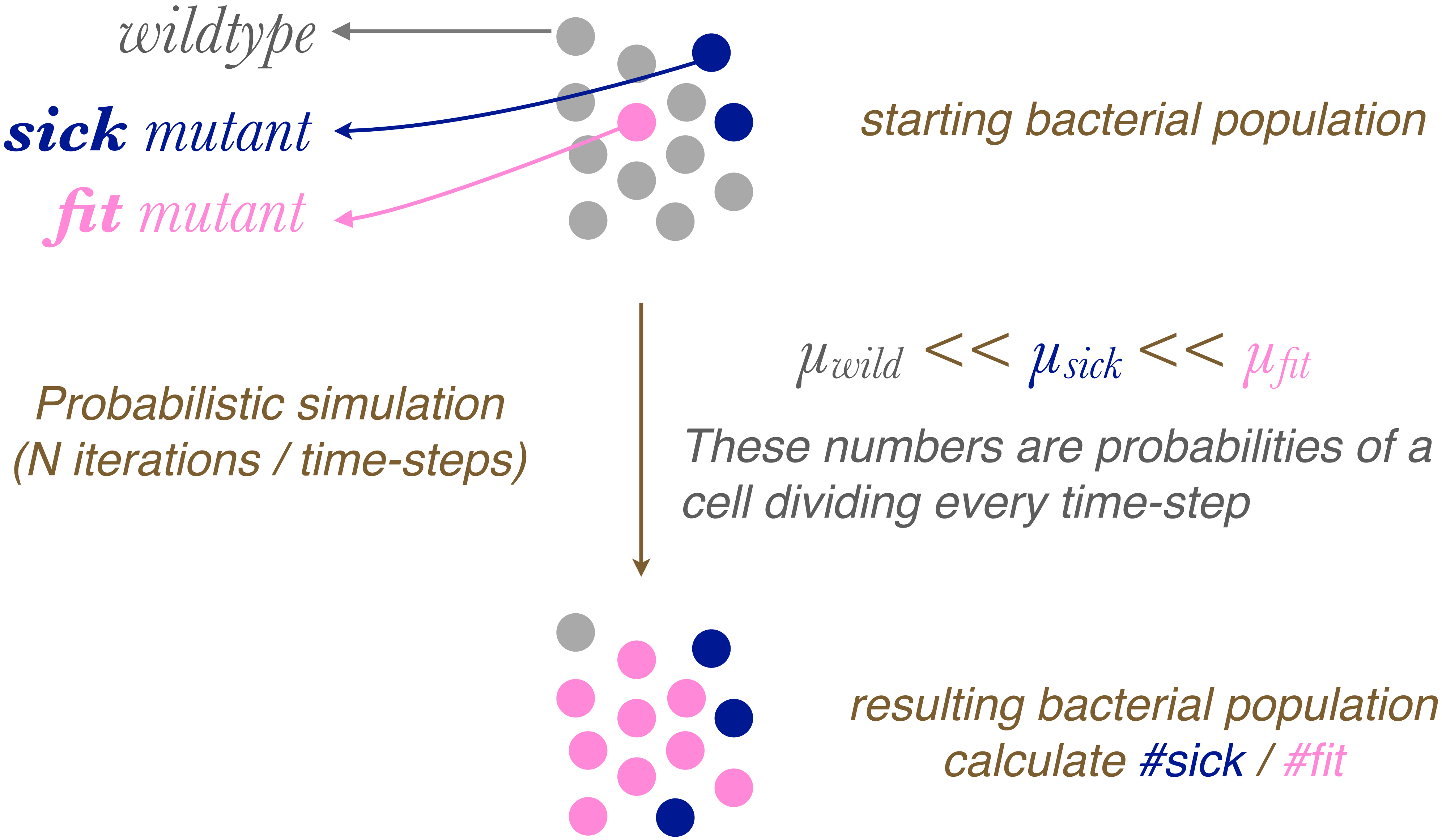
wildtype
sick mutant
fit mutant
starting bacterial population
Probabilistic simulation
(N iterations / time-steps)
$\mu_{wild} << \mu_{sick} << \mu_{fit}$
These numbers are probabilities of a cell dividing every time-step
resulting bacterial population
calculate #sick / #fit

Figure S7:

Population structure of evolving populations (part II).

(A) Results of the two trials represented as boxplots of the ratios of OD_{600} in 20-kan to OD_{600} in 4-kan for isolates falling into the three groups (see inset, Figure 2B, 8-kan 1). Data for the three types of isolates from stationary phase 4-kan and 8-kan populations are plotted. The three group types are represented by different shades of grey (see inset copied from Figure 2B for definition of groups) and the populations that they were derived from are indicated on the x-axis. The two different replicates are indicated above. For simplicity, groups are excluded if isolates of that type weren't picked up. Shown here are 'distribution' representations by box plots. The box represents the interquartile range and the line within the box indicates the median value. The maximum and the minimum value (1.5 times the interquartile range), excluding the outliers, is represented by the whiskers. The points represent the outliers. As the ability to grow in 20-kan equals the ability to grow in 4-kan, as it does for the group-1 colonies (which are most likely ATP synthase mutants), the mean in the box plot of ratios should approach 1. While if the ability to grow in 20-kan declines sharply with respect to growth in 4-kan, then the mean in the box-plots should decrease as it does for the group-3 colonies – although these are fit, tolerant mutants with a higher MIC.

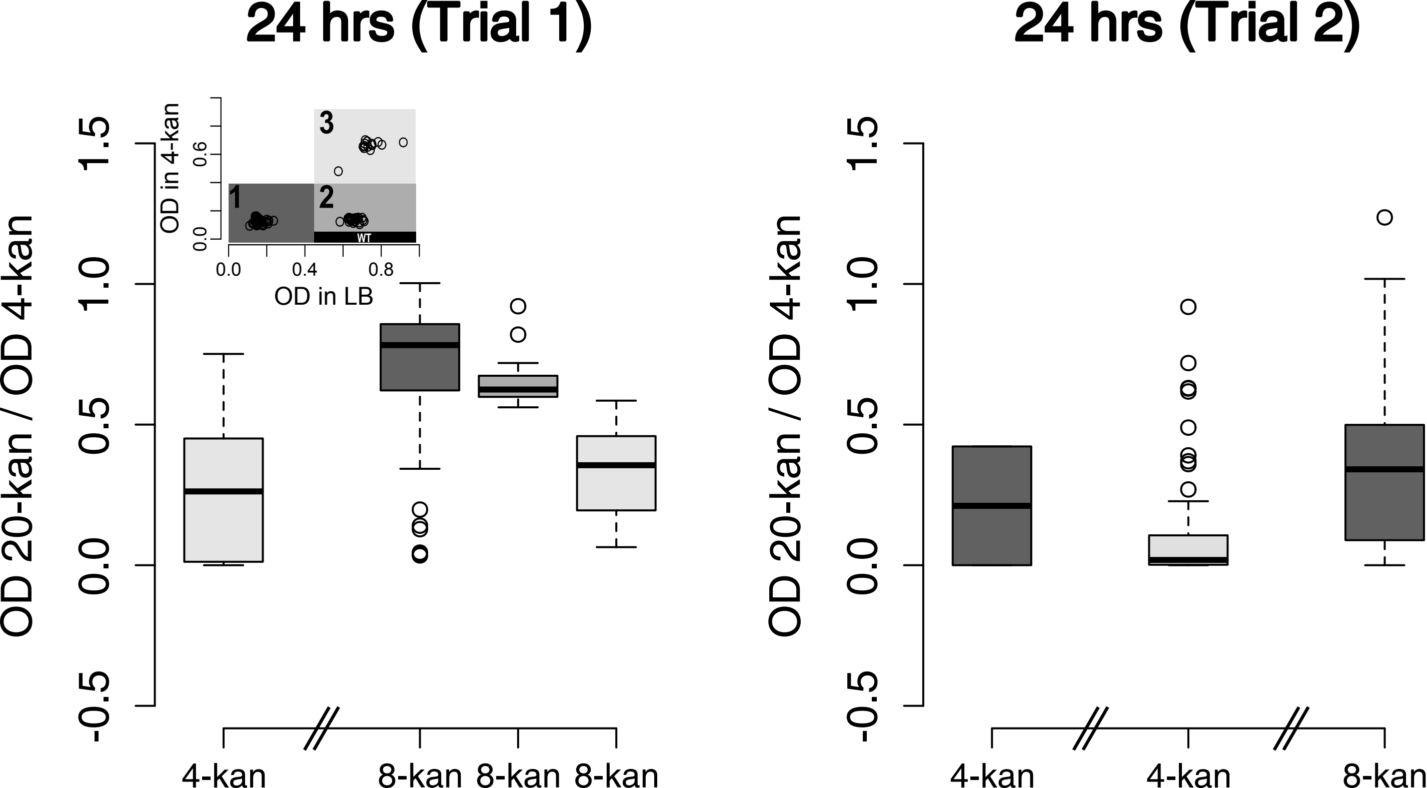
24 hrs (Trial 1)
24 hrs (Trial 2)
OD 20-kan / OD 4-kan
1.5
1.0
0.5
0.0
-0.5
OD in 4-kan
OD in LB
0.0
0.6
0.0
0.4
0.8
1
2
3
WT
4-kan
8-kan
8-kan
8-kan
4-kan
4-kan
8-kan

Figure S8:

Analysis of isolates, derived from the third passage of 4-kan populations, containing the *rho*/*wecA* G>T intergenic region (IGR) mutation.

(A) Presence (red)/ absence (black) heatmaps of mutations in isolates, derived from the two replicates of 4-kan populations in the third passage, confirmed by Sanger sequencing. Isolates are represented along the horizontal axis and mutations along the vertical axis. Isolates 2 and 4 also have the *rho*/*wecA* IGR mutation. In the first replicate the mutation in *acrE* does not fix (Figure 3A). This can be seen here as well. Also from the Sanger sequencing of the isolates, since the $FusA^{P610T}$ mutation is present in both backgrounds, i.e. isolates with or without the mutation in *acrE*, this scenario suggests that the $FusA^{P610T}$ mutation appeared independently in both backgrounds and explains the maintenance of $AcrE^{R334L}$ levels in the first replicate. (B) Kanamycin MIC boxplots (n=8) for the 10 isolates (MICs of WT-FRT and isolate 1 are copied from Figure 3B). These are 'distribution' representations by box plots. The box represents the interquartile range and the line within the box indicates the median value. The maximum and the minimum value (1.5 times the interquartile range), excluding the outliers, is represented by the whiskers. The points represent the outliers. (C) Kanamycin MIC curves for *wildtype* (black), $FusA^{P610T}$ (red), isolates 2 (green) and 4 (blue) with the IGR *rho*/*wecA* mutation present along with $FusA^{P610T}$. These curves most likely imply that the *rho*/*wecA* mutation is another hitch-hiker and does not contribute to tolerance (error bars: standard deviation derived from OD_{600} values of three technical replicates). (D) RT-PCR does not reveal a clear regulatory effect of the *rho/*wecA intergenic mutation on downstream genes. Relative C_t values, of three genes downstream of the *rho*/*wecA* IGR mutation (*wecA*, *rffH* and *rffM*) and *ffh* a randomly picked gene elsewhere on the *E. coli* genome, for $FusA^{P610T}$ (FA) and $FusA^{P610T}$ + IGR *rho*/*wecA* mutant (RW). C_t values are relative to those of 16S rRNA. Darker colours represent samples taken from mid-exponential (ME) phase and lighter colours represent stationary (S) phase samples. Black and green represent growths followed in plain LB whereas red and blue represent growth followed in 4-kan (error bars represent standard deviation of the combination of two biological replicates and their three technical replicates). The differences in the expression of these genes across the FA and RW strains is not significant regardless of the phase and the presence of kanamycin (although a difference does exist in expression in the presence versus absence of kanamycin within a strain). The inset shows the position of the three genes selected for analysis in the operon downstream the intergenic mutation. The following table shows fold change in relative (to 16S) C_t values in the RR strain compared to FA (in absence of kanamycin):

Fold change in C_t values RR (vs FA)	*ffh*	*rfe*	*rffH*	*rffM*
ME	0.9979	1.0159	1.0027	0.9904
S	1.0094	1.0021	1.0221	1.0397

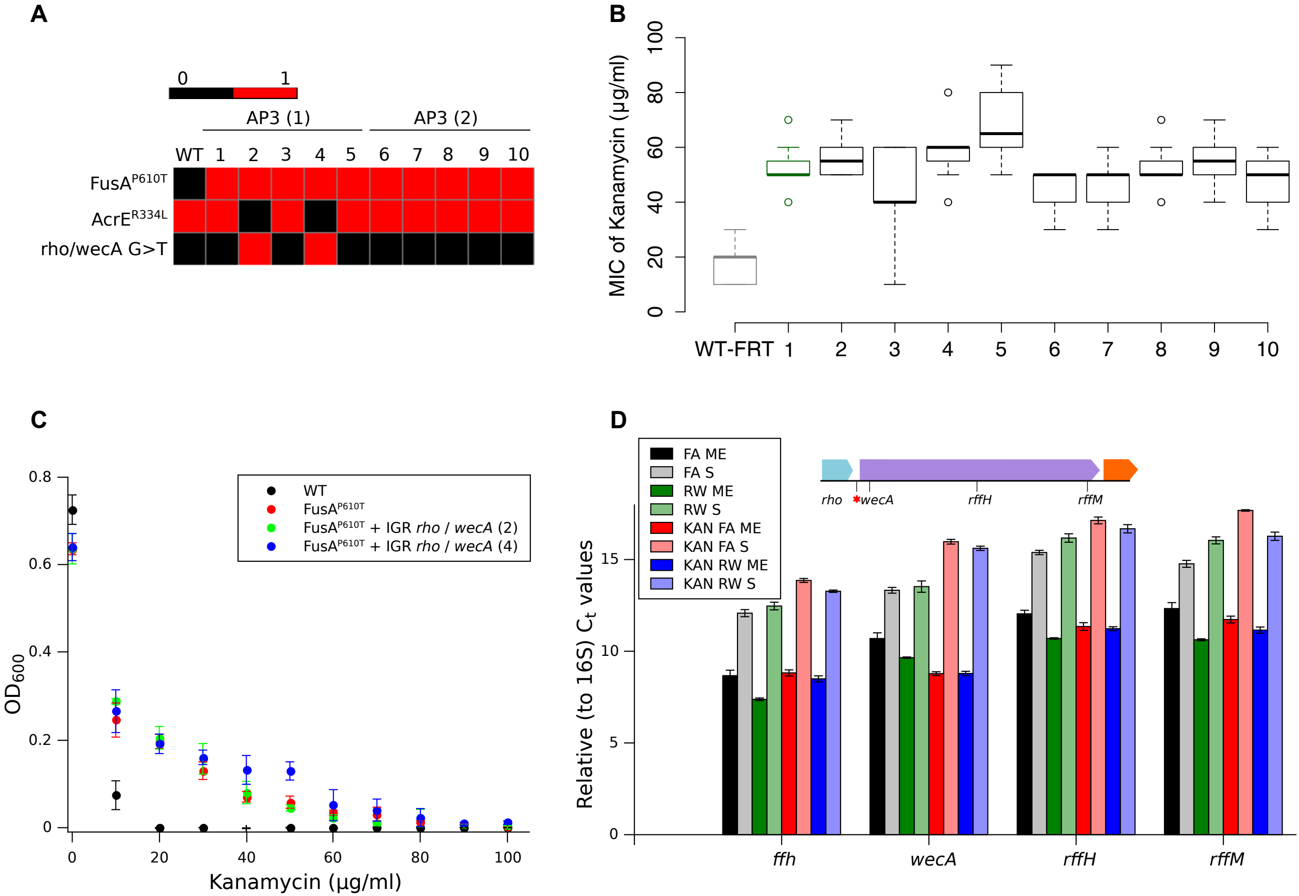

A
0
1
AP3 (1)
AP3 (2)
WT 1 2 3 4 5 6 7 8 9 10
FusA P610T
AcrE R334L
rho/wecA G>T
B
MIC of Kanamycin (µg/ml)
WT-FRT 1 2 3 4 5 6 7 8 9 10
C
OD600
Kanamycin (µg/ml)
WT
FusA P610T
FusA P610T + IGR rho / wecA (2)
FusA P610T + IGR rho / wecA (4)
D
FA ME
FA S
RW ME
RW S
KAN FA ME
KAN FA S
KAN RW ME
KAN RW S
rho
wecA
rffH
rffM
Relative (to 16S) Ct values
ffh
wecA
rffH
rffM

Figure S9:

Location of the tolerance conferring mutations in EF-G.

Protein feature view of EF-G from *Thermus thermophilus*, with the region at the end of domain IV highlighted with a blue box. The three mutations ($FusA^{P610T}$, $FusA^{P610L}$ and $FusA^{A608E}$) are in this region. The crystal structure of EF-G from *Thermus* (1EFG) is shown below the protein feature view with the residues corresponding to *E. coli* P610 and A608 highlighted with a blue circle and represented as spheres.

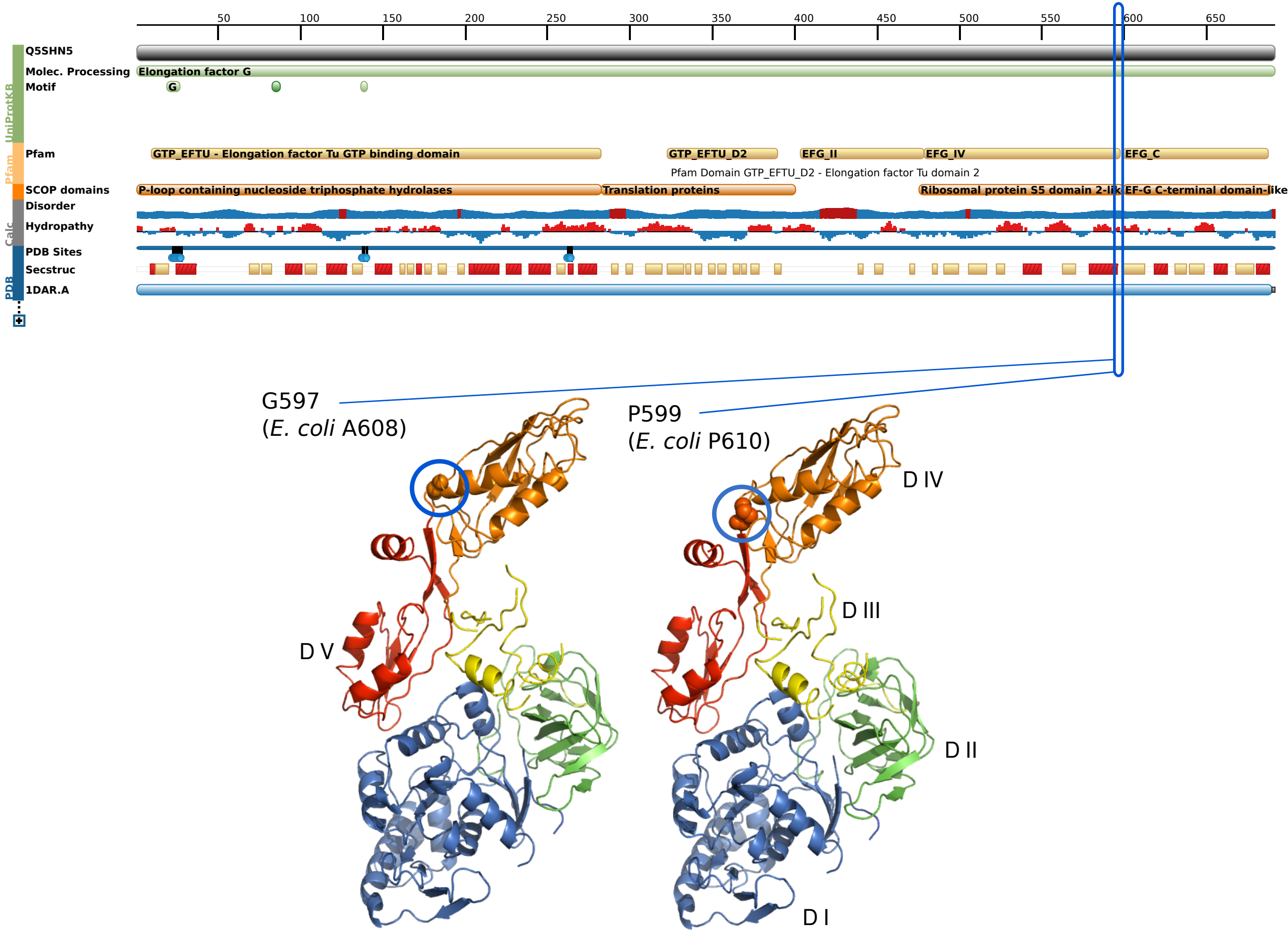

50
100
150
200
250
300
350
400
450
500
550
600
650
UniProtKB
Pfam
Calc
PDB
Q5SHN5
Molec. Processing
Motif
Elongation factor G
G
Pfam
GTP_EFTU - Elongation factor Tu GTP binding domain
GTP_EFTU_D2
EFG_II
EFG_IV
EFG_C
Pfam Domain GTP_EFTU_D2 - Elongation factor Tu domain 2
SCOP domains
P-loop containing nucleoside triphosphate hydrolases
Translation proteins
Ribosomal protein S5 domain 2-lik
EF-G C-terminal domain-like
Disorder
Hydropathy
PDB Sites
Secstruc
1DAR.A
G597
(E. coli A608)
P599
(E. coli P610)
D IV
D III
D V
D II
D I

Figure S10:

The FusA^{P610T} mutation confers tolerance to kanamycin without conferring a cost in the absence of the antibiotic (similar experiments in Figure 3).

(A) Spotting of 10-fold dilutions of overnight grown wildtype (WT) and FusA^{P610T}-FRT cultures on LB agar and LB agar containing 4, 15, 25 and 50-kan (indicated on the right). Temperature of incubation after spotting is indicated on the top along with the dilutions of the culture (UD represents undiluted culture and 1-7 represent serial 10-fold dilutions). The FusA^{P610T} mutation confers tolerance, and does not adversely affect growth, even at higher temperatures. (B) Competition between the *wildtype* (without the FRT site near *fusA* as opposed to *wt::*FRT competed in Figure 3E) and FusA^{P610T}-FRT strains. Bold colours represent the competition experiment, while the light colours represent the mock competition experiment where either strains were grown separately and mixed prior to plating. The FusA^{P610T}-FRT strain can compete well even with the *wildtype* without the FRT site near the *fusA* gene. (C) Promoter activities (normalised to fall between 0 and 1) of P_{BAD} promoter (GFP^{mut2} induction) in WT-FRT versus FusA^{P610T}-FRT for two different concentrations of inducer arabinose, (1mM – 1 Ara; 10mM – 10 Ara) and uninduced control (UI), in the presence of 10 µg/ml Kanamycin. The box represents time after induction where promoter activity peaks in the culture not treated with kanamycin (Figure 3F). Around OD_{600} 0.5, along with the inducer, kanamycin to a final concentration of 10 µg/ml was also added (error bars indicate standard deviation for four replicates; for induction in absence of kanamycin see Figure 3F). Shown are time-points after addition of inducer and kanamycin. (D) Growth curves of the strains after addition of inducer arabinose in 0-kan (upper panel) and 10-kan (lower panel). Colour scheme used in (C) is retained in these plots. Only time-points after induction are shown. The box represents time after induction where promoter activity peaks in the culture not treated with kanamycin (Figure 3F).

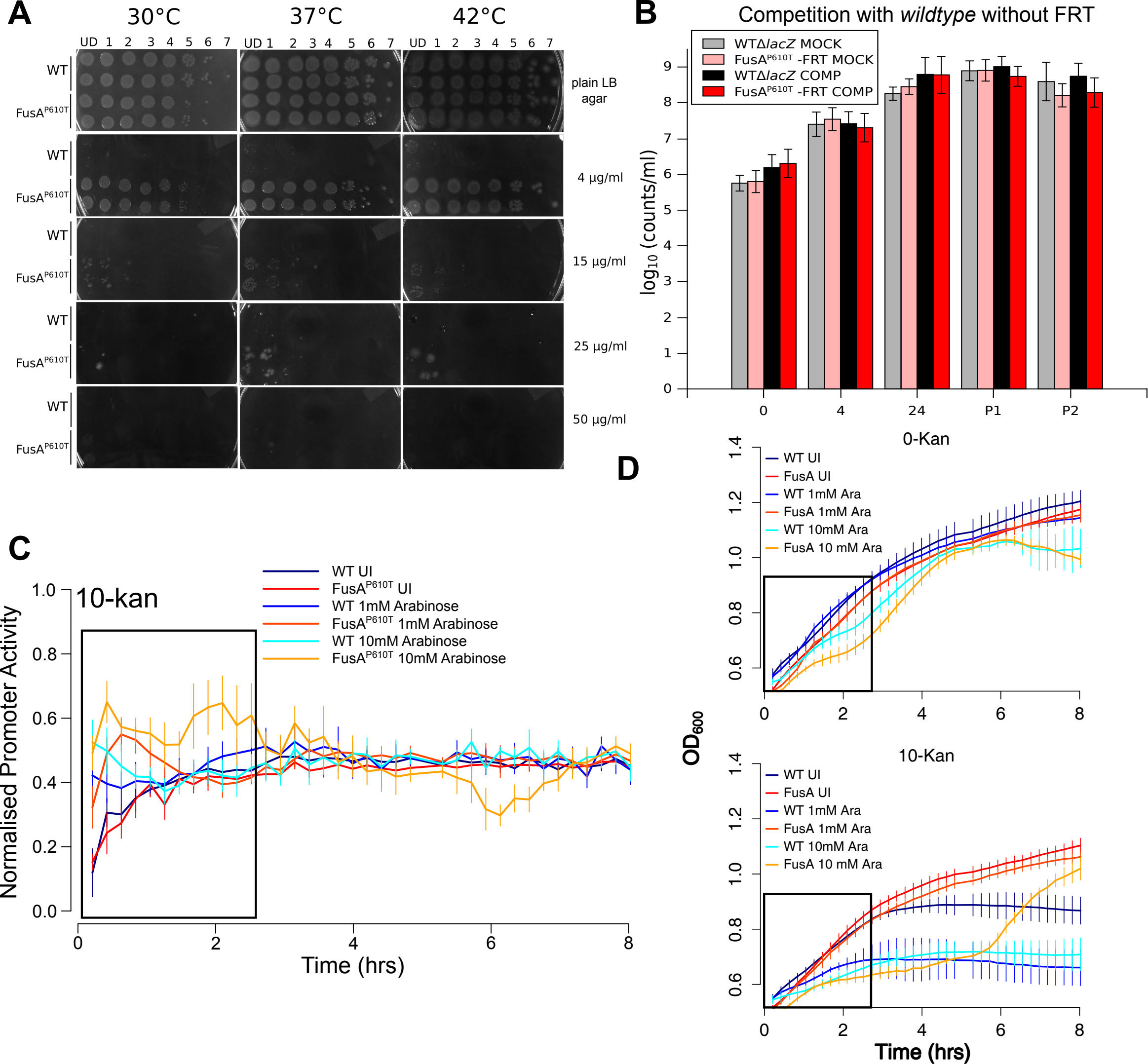

A
30°C
37°C
42°C
UD 1 2 3 4 5 6 7
WT
FusA P610T
plain LB agar
4 µg/ml
15 µg/ml
25 µg/ml
50 µg/ml
B
Competition with wildtype without FRT
WTΔlacZ MOCK
FusA P610T -FRT MOCK
WTΔlacZ COMP
FusA P610T -FRT COMP
log10 (counts/ml)
0
4
24
P1
P2
C
10-kan
WT UI
FusA P610T UI
WT 1mM Arabinose
FusA P610T 1mM Arabinose
WT 10mM Arabinose
FusA P610T 10mM Arabinose
Normalised Promoter Activity
Time (hrs)
D
0-Kan
10-Kan
WT UI
FusA UI
WT 1mM Ara
FusA 1mM Ara
WT 10mM Ara
FusA 10 mM Ara
OD600
Time (hrs)

Figure S11:

Location of tolerance conferring deletions in genes encoding subunits of the ATP synthase machinery. Protein feature view of *atpA* (A) and *atpG* (B). The amino acid sequence of the deleted regions is displayed above the protein feature view. The deleted regions are shown in red in the crystal structures on the right (3OAA.A and 3OAA.G). The deleted region shown in the crystal structure of *atpG* corresponds to the deletion length in the *atpGΔ108bp* mutation. (C) The deletions in *atpA* and *atpG* are both shown in red and are nearby in three-dimensional space.

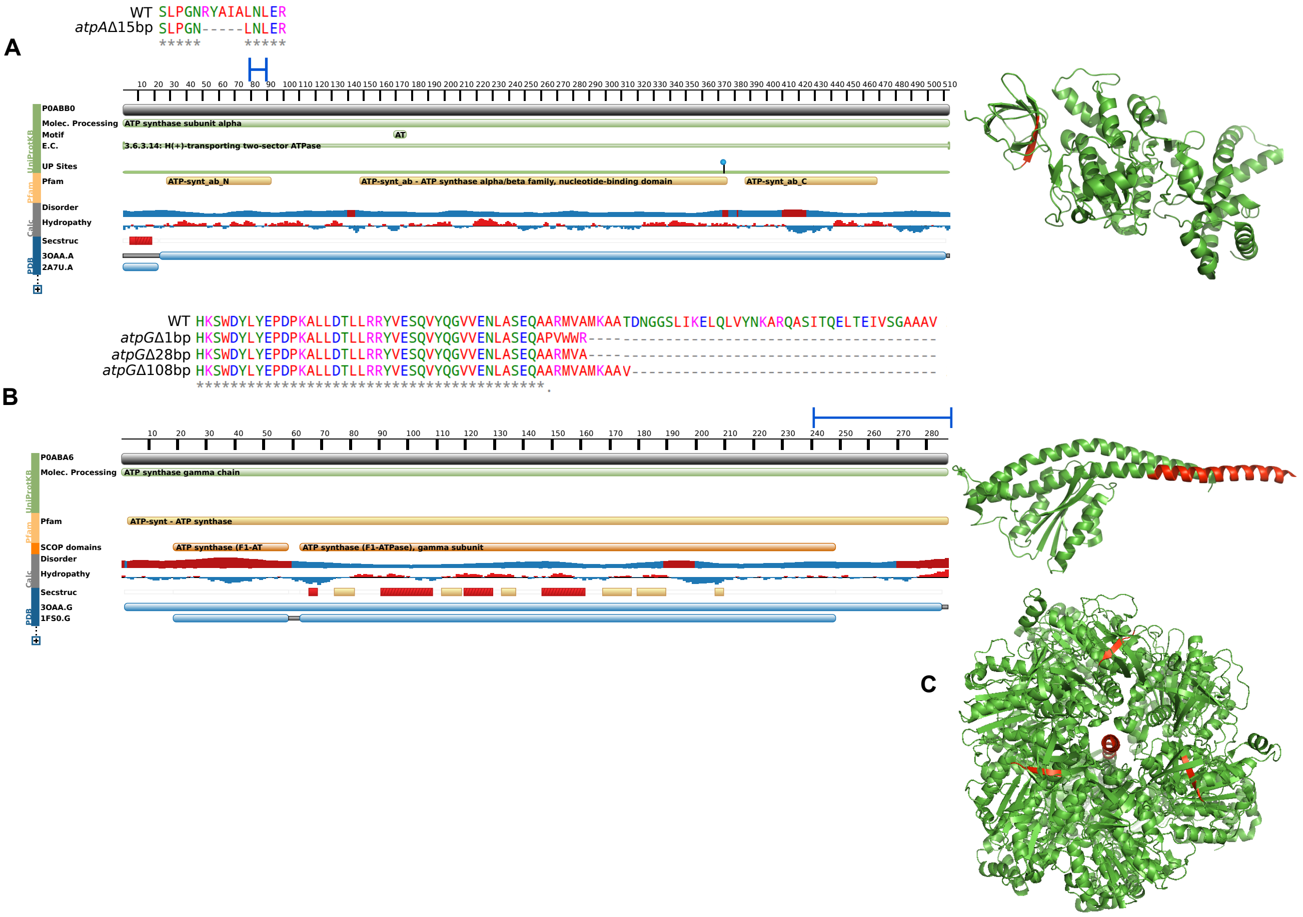
A
WT SLPGNRYAIALNLER
atpAΔ15bp SLPGN-----LNLER
P0ABB0
Molec. Processing
ATP synthase subunit alpha
Motif
AT
E.C.
3.6.3.14: H(+)-transporting two-sector ATPase
UP Sites
Pfam
ATP-synt_ab_N
ATP-synt_ab - ATP synthase alpha/beta family, nucleotide-binding domain
ATP-synt_ab_C
Disorder
Hydropathy
Secstruc
3OAA.A
2A7U.A
UniProtKB
Pfam
Calc
PDB
B
WT HKSWDYLYEPDPKALLDTLLRRYVESQVYQGVVENLASEQAARMVAMKAATDNGGSLIKELQLVYNKARQASITQELTEIVSGAAAV
atpGΔ1bp HKSWDYLYEPDPKALLDTLLRRYVESQVYQGVVENLASEQAPVWWR
atpGΔ28bp HKSWDYLYEPDPKALLDTLLRRYVESQVYQGVVENLASEQAARMVA
atpGΔ108bp HKSWDYLYEPDPKALLDTLLRRYVESQVYQGVVENLASEQAARMVAMKAAV
P0ABA6
Molec. Processing
ATP synthase gamma chain
Pfam
ATP-synt - ATP synthase
SCOP domains
ATP synthase (F1-AT
ATP synthase (F1-ATPase), gamma subunit
Disorder
Hydropathy
Secstruc
3OAA.G
1FS0.G
C

Figure S12:

Residues A608 (A) and P610 (B) in *E. coli* FusA are well conserved across sequenced bacterial genomes.

Frequency of occurrence of residues at the indicated position are plotted. NH indicates no-hit, - indicates residue deletion in the target genome and single letter codes for amino acids indicate the substituted residue at that position. Residues indicated in red are the mutated residues seen in our study.

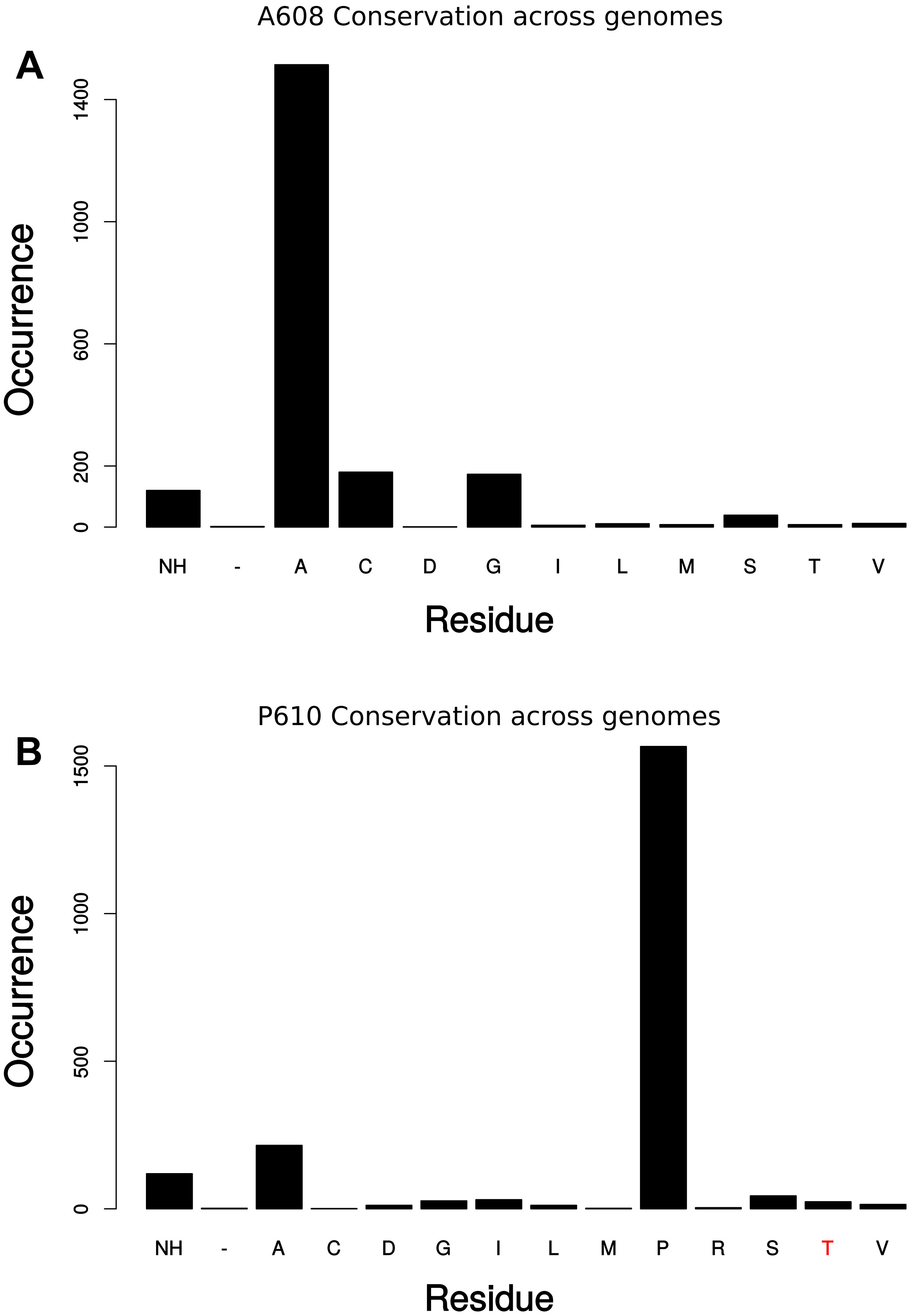
A608 Conservation across genomes
A
Occurrence
0
200
600
1000
1400
NH
-
A
C
D
G
I
L
M
S
T
V
Residue
P610 Conservation across genomes
B
Occurrence
0
500
1000
1500
NH
-
A
C
D
G
I
L
M
P
R
S
T
V
Residue

Figure S13:

Resistance of the FusA^{P610T} mutation across a panel of aminoglycosides.

We studied the tolerance of FusA^{P610T} mutation to different aminoglycosides, representing streptamine-containing and 2-DOS-containing groups – using the FusA^{P610T}-FRT strain. In this regard, streptomycin (streptamine group); and kanamycin, gentamicin, neomycin, paromomycin, apramycin and hygromycin (2-DOS group) were tested. The 2-DOS aminoglycosides tested can further be subdivided as 4,5-disubstituted (neomycin, paromomycin, apramycin and hygromycin) and 4,6-disubstituted (kanamycin and gentamicin) 2-DOS derivatives. All the tested aminoglycosides, with the exception of apramycin and hygromycin, are paromomine derivatives. The FusA^{P610T} strain tolerates all 2-DOS aminoglycosides (A and B), but does not tolerate streptomycin (C; black bars indicate wildtype with FRT site near *fusA* and red bars indicate FusA^{P610T}-FRT strain; error bars indicate standard deviation of 8 replicates). The approximate fold tolerances are: 3-fold for kanamycin, neomycin and paromomycin, less than 2-fold for gentamicin, 2-fold for hygromycin and 5-fold for apramycin. The MIC changes in the FusA^{P610T} mutant are in line with the explanation that translocation blockade of ribosomes is the basis for the bactericidal action of the 2-DOS aminoglycosides [1]. Apramycin and hygromycin have an unusual fused ring structure. While the fused ring aminoglycosides like apramycin do not cause miscoding and mainly affect translocation, the other 2-DOS aminoglycosides induce miscoding along with translocation blockade [1]. Also, paromomycin has a stronger inhibitory effect on the translocation of ribosomes on the first codon whereas gentamicin disrupts translocation across all codons [1]; most likely this is reflected in the lower MICs of gentamicin. On the other hand, the fold increase in resistance provided by the FusA^{P610T} mutation is greater for paromomycin than gentamicin. Between gentamicin and paromomycin, inhibition of the pre-translocational state is greater in the case of gentamicin[1 (consider Figures 2E & F in this reference)]. Assuming that the FusA^{P610T} mutation reverses resistance via its action on ribosomes blocked in the post-translocation step, the lower fold change in case of gentamicin makes sense. Streptomycin, however, binds to a region distinct but nearby to that bound by the 2-DOS aminoglycosides, and by a different mechanism induces miscoding [2].

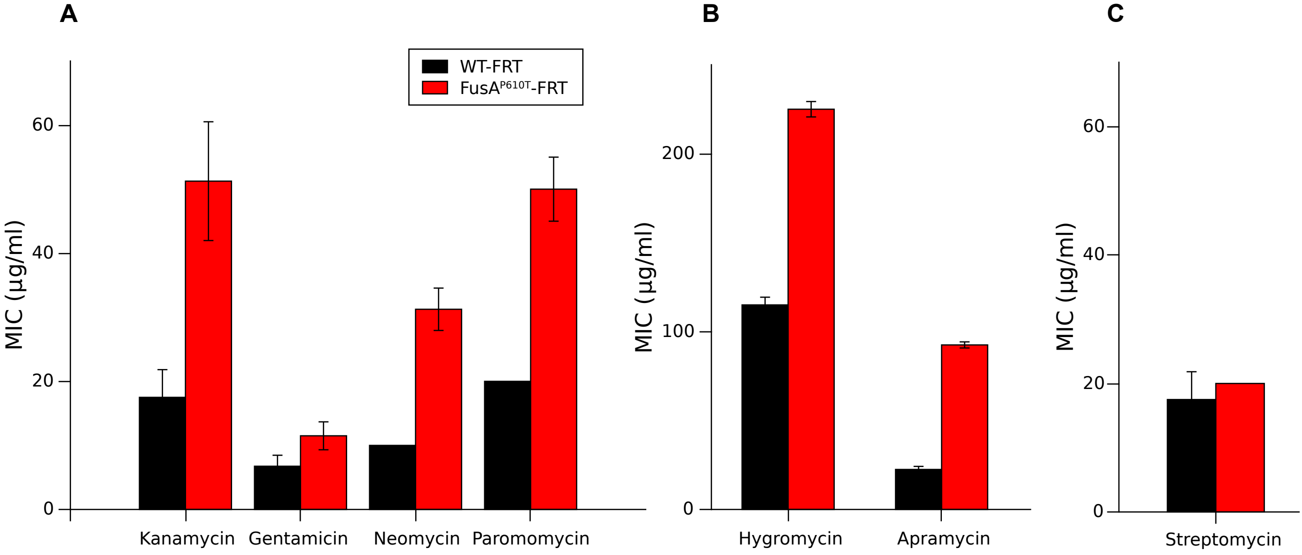

A
B
C
WT-FRT
FusA^{P610T}-FRT
MIC (µg/ml)
0
20
40
60
Kanamycin
Gentamicin
Neomycin
Paromomycin
MIC (µg/ml)
0
100
200
Hygromycin
Apramycin
MIC (µg/ml)
0
20
40
60
Streptomycin

Figure S14:

Temperature sensitive mutation in RRF does not interact with the kanamycin tolerance conferring mutation in *fusA*.

EF-G, along with the ribosome recycling factor (RRF), assists in dissociation of stop codon stalled ribosome complexes [3]. 2-DOS aminoglycosides, apart from inducing mistranslation and translocation blockade, could also affect ribosome recycling. We checked for rescue of a temperature sensitive mutation in the ribosome recycling factor (RRF, *frr*^{ts}), by the P610T mutation in *fusA*. The *frr*^{ts} allele was transferred into the FusA^{P610T} strain from LJ14 [3] using P1 transduction. Though it is not clear that the temperature sensitive mutation in RRF has anything to do with aminoglycoside activity, our data show that this RRF residue and the P610 mutation in EF-G are probably uncoupled. This figure shows kanamycin MIC curves (3 replicates), with incubation temperature mentioned above (34°: non-restrictive temperature, 37°: slightly restrictive temperature and 42°C: restrictive temperature; error bars are standard deviation from three replicates).

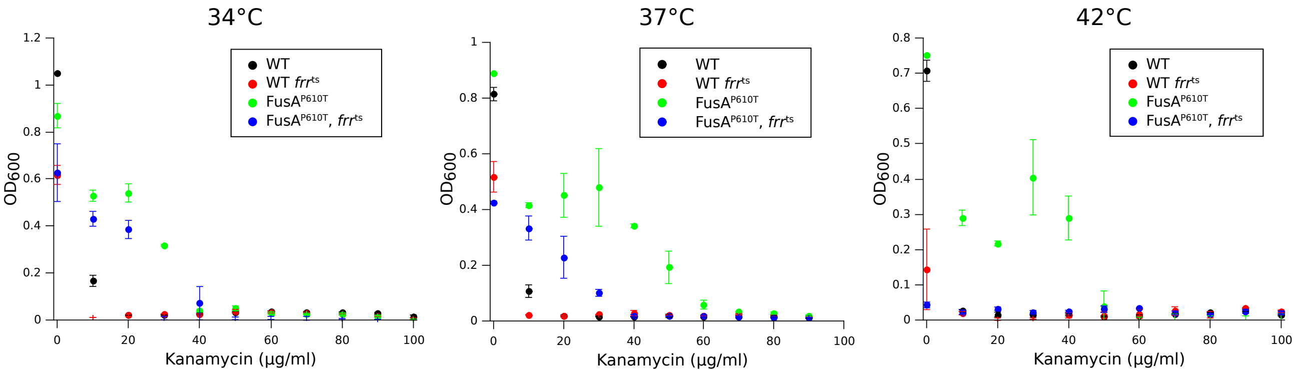

34°C
37°C
42°C
OD600
Kanamycin (μg/ml)
WT
WT frr^ts
FusA^P610T
FusA^P610T, frr^ts

Figure S15:

Tolerance characteristics of the ATP Synthase mutants.

(A) Normalised MIC curves (N = ODconc. [C] / ODconc. [0]) of *wildtype* (WT-FRT, black), $FusA^{P610T}$-FRT (red) and *atpGΔ108bp* (green). OD_{600} for all concentrations is plotted as a ratio to the OD_{600} at 0-kan. As was indicated in Figure S7, the growth of the ATP synthase mutants declines gradually with increasing kanamycin concentrations in comparison with either the *wildtype* or the $FusA^{P610T}$ mutant. (B) Actual MIC curves for comparison (error bars indicate standard deviation of 8-replicates).

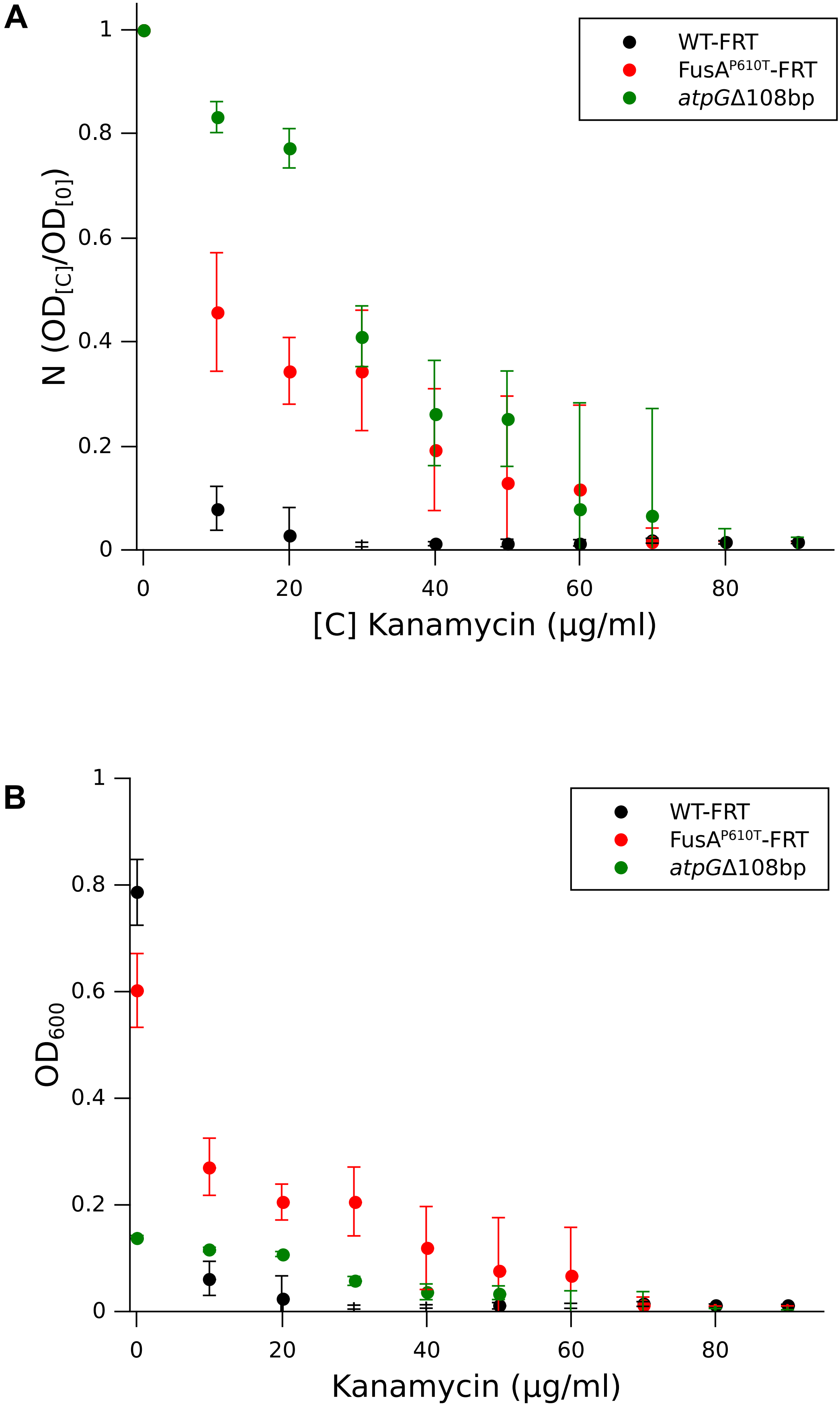
A
1
0.8
0.6
0.4
0.2
0
N (OD[C]/OD[0])
0
20
40
60
80
[C] Kanamycin (µg/ml)
WT-FRT
FusAP610T-FRT
atpGΔ108bp
B
1
0.8
0.6
0.4
0.2
0
OD600
0
20
40
60
80
Kanamycin (µg/ml)
WT-FRT
FusAP610T-FRT
atpGΔ108bp

References:

1. Tsai, A., Uemura, S., Johansson, M., et al. 2013, The impact of aminoglycosides on the dynamics of translation elongation. *Cell Rep.*, **3**, 497–508.
2. Demirci, H., Murphy, F., 4th, Murphy, E., Gregory, S. T., Dahlberg, A. E., and Jogl, G. 2013, A structural basis for streptomycin-induced misreading of the genetic code. *Nat. Commun.*, **4**, 1355.
3. Rao, A. R., and Varshney, U. 2001, Specific interaction between the ribosome recycling factor and the elongation factor G from Mycobacterium tuberculosis mediates peptidyl-tRNA release and ribosome recycling in Escherichia coli. *EMBO J.*, **20**, 2977–86.

Table S1: List of mutations in 4-kan populations

Gene	Description	Coordinate	Mutation	Change in residue	References
acrE →	Component of: AcrEF-TolC multidrug efflux transport system (mutation is in C-terminal region essential for function)	3412886	G→T	R334L (CGC→CTC)	[1]
fusA ←	Translational elongation factor – G, catalyses translocation. Mutations known to give kanamycin resistance.	3469709	G→T	P610T (CCA→ACA)	[2,3]
rho/wecA	transcription termination factor/UDP-GlcNAc:undecaprenylphosphate GlcNAc-1-phosphate transferase	3965775	G→T	intergenic (+76/-164)	
rrlA	23S ribosomal RNA of rrnA operon	4037405	T→C	noncoding (1864/2905 nt)	
rrlA	23S ribosomal RNA of rrnA operon	4037438	G→A	noncoding (1897/2905 nt)	
cpxA ←	Sensory histidine kinase	4102346	A→T	F218Y (TTC→TAC)	[4,5]

References:

1. Ge, Q., Yamada, Y., and Zgurskaya, H. 2009, The C-terminal domain of AcrA is essential for the assembly and function of the multidrug efflux pump AcrAB-TolC. *J. Bacteriol.*, **191**, 4365–71.
2. Hou, Y., Lin, Y. P., Sharer, J. D., and March, P. E. 1994, In vivo selection of conditional-lethal mutations in the gene encoding elongation factor G of Escherichia coli. *J. Bacteriol.*, **176**, 123–9.
3. Lázár, V., Pal Singh, G., Spohn, R., et al. 2013, Bacterial evolution of antibiotic hypersensitivity. *Mol. Syst. Biol.*, **9**, 700.
4. Kohanski, M. A., Dwyer, D. J., Hayete, B., Lawrence, C. A., and Collins, J. J. 2007, A common mechanism of cellular death induced by bactericidal antibiotics. *Cell*, **130**, 797–810.
5. Mahoney, T. F., and Silhavy, T. J. 2013, The Cpx stress response confers resistance to some, but not all, bactericidal antibiotics. *J. Bacteriol.*, **195**, 1869–74.

Table S2: Coverages of ribosomal genes.

E. coli has seven genes coding for 16S-rRNA (*rrsA-E, G-H*). Each gene is 1541 nucleotides long.
In the following table:
(A) Total number of reads mapping to any of the seven 16S-rRNA genes.
(B) Average coverage per gene (averaged across all *E. coli* genes) in the sample.
The last column represents fold change in coverage per 16S-rRNA gene. This value is obtained by first normalising the number of reads mapping to rrs (A) by the length of the gene (1541). Next to get fold change in coverage, this value was divided by the average coverage per gene obtained in that sample (B). Since there are 7 copies of the gene, this fold change in coverage seen is around 7. This value is then divided by 7 to get fold change in coverage per rrs gene.

4-kan	Reads mapping to rrs (A)	Average coverage per gene (B)	Fold change in coverage per rrs gene
15 hrs rep 1	4328172	358.074596286	1.1205476302
15 hrs rep 2	2946609	253.2221948134	1.078748149
17 hrs rep 1	1966994	172.5495069428	1.0567898509
17 hrs rep 2	2975612	252.3331805142	1.093204123
19 hrs rep 1	2127600	191.9892368062	1.0273357959
19 hrs rep 2	2867358	247.2703063478	1.0750020042
21 hrs rep 1	3053929	268.7193309623	1.0535601798
21 hrs rep 2	3819714	335.4467956667	1.0556173511
23 hrs rep 1	1345196	118.3240212752	1.0539304874
23 hrs rep 2	2609927	228.7284257846	1.0578096888
AP1 rep 1	3803828	340.9780289431	1.0341744315
AP1 rep 2	7128459	612.7730107729	1.0784384127
AP2 rep 1	1415075	125.5188404904	1.0451289094
AP2 rep 2	4192742	364.2996081998	1.0669369506
AP3 rep 1	838881	75.4764077531	1.0303587352
AP3 rep 2	2176118	192.7329414183	1.0467086497
AP4 rep 1	5430735	494.0638787656	1.0190015134
AP4 rep 2	5325788	471.4735725556	1.047190857
AP5 rep 1	5090948	444.3785432428	1.0620498488
AP5 rep 2	7942073	710.0356862169	1.0369385467
0-kan			
ME rep 1	2233130	186.8217641047	1.1081176148
ME rep 2	3713898	311.7248486099	1.1044800368
S rep 1	2025765	191.2235514425	0.9820803124
S rep 2	1444688	131.0959766049	1.0216073845
P1 rep 1	4263156	382.7242154302	1.0326296831
P1 rep 2	4967070	444.5640670606	1.0357745716
P2 rep 1	5489457	502.0420141363	1.013651449
P2 rep 2	2876456	259.0702581753	1.029294136
P3 rep 1	3747570	337.0892387021	1.0306333384
P3 rep 2	3286313	301.5926336141	1.0101538336
P4 rep 1	2545997	241.0281552775	0.9792406465
P4 rep 2	4334384	389.5779183791	1.0314124588
P5 rep 1	2638934	236.9938573594	1.0322639489
P5 rep 2	1728135	155.9198716319	1.0274850671
8-kan P0			
B	2869601	257.6083331969	1.0326684975
C	2886637	258.8755684399	1.0337140752
D	3319891	282.1778552119	1.0906872043
E	4442635	394.692320105	1.0434731511
F	2986720	269.3013403418	1.0281472458
G	2911581	266.2388294082	1.0138105418
H	3286277	276.0285148477	1.103696181
I	3176409	283.1969818073	1.0397935488
J	3441525	318.8505551472	1.0006059303
K	3578291	328.4007161674	1.010115143

L	2950641	256.5608602282	1.0661671346
8-kan P1			
N	1954659	234.2548515055	0.773538138
O	2903762	258.9025566759	1.0397381987
P	2840254	260.671702661	1.0100959103
Q	3496998	339.2051491581	0.9557235089
R	3033832	328.337603293	0.8565845273
S	2181833	235.2818418959	0.859671276
T	2322143	206.773604527	1.0411017662
U	3217441	320.621504585	0.9302876707
V	2439142	250.1907846603	0.9037849322
W	2087809	188.5260461146	1.0266412909
X	2540467	228.1923927085	1.0320760902
0-kan P0			
A	4605034	418.139792255	1.020964551
0-kan P1			
M	3629029	336.5745277759	0.9995591863

Table S3: List of mutations in isolates from 8-kan populations

Gene	Description	Coordinate	Mutation	Change in residue	
yajG ←		453005	G→T	I129I (ATC→ATA)	
ybfQ → / → ybfL		736003	A→G	intergenic (+96/-45)	
topA →	DNA topoisomerase I (ω protein)	1329610	C→T	S180L (TCG→TTG)	
gatC	Contains a PTS Enzyme IIC domain. (Insertion similar to the one seen in Freddolino PL et. al. 2012)	2171384	.→C	coding (917/1356 nt)	[1]
yfiP →		2717619	A→T	P125P (CCA→CCT)	
rpoD →	RNA polymerase sigma 70	3211850	T→A	L261Q (CTG→CAG)	
acrE →	Component of: AcrEF-TolC multidrug efflux transport system (mutation is in C-terminal region essential for function)	3412886	G→T	R334L (CGC→CTC)	[2]
fusA ←	Translational elongation factor – G, catalyses translocation. Mutations known to give kanamycin resistance.	3469708	G→A	P610L (CCA→CTA)	[3,4]
fusA ←	Translational elongation factor – G, catalyses translocation. Mutations known to give kanamycin resistance.	3469714	G→T	A608E (GCG→GAG)	[3,4]
yhgA →		3541595	G→A	W136* (TGG→TAG)	
atpG ←	Gamma subunit ATP synthase F1 complex	3915437	Δ108 bp	coding (745-852/864 nt)	[4]
atpG ←	Gamma subunit ATP synthase F1 complex	3915527	Δ28 bp	coding (735-762/864 nt)	[4]
atpG ←	Gamma subunit ATP synthase F1 complex	3915566	Δ1 bp	coding (723/864 nt)	[4]

atpA ←	Alpha subunit ATP synthase F1 complex	3917692	Δ15 bp	coding (175-189/1542 nt)	[4]
cyaA →	Adenylate cyclase	3990973	A→T	N600Y (AAC→TAC)	
cpxA ←	Sensory histidine kinase	4102346	A→T	F218Y (TTC→TAC)	[5,6]
oxyR →	Bifunctional regulatory protein sensor for oxidative stress	4157045	G→T	R178L (CGC→CTC)	

References:

1. Freddolino, P. L., Amini, S., and Tavazoie, S. 2012, Newly identified genetic variations in common Escherichia coli MG1655 stock cultures. *J. Bacteriol.*, **194**, 303–6.
2. Ge, Q., Yamada, Y., and Zgurskaya, H. 2009, The C-terminal domain of AcrA is essential for the assembly and function of the multidrug efflux pump AcrAB-TolC. *J. Bacteriol.*, **191**, 4365–71.
3. Hou, Y., Lin, Y. P., Sharer, J. D., and March, P. E. 1994, In vivo selection of conditional-lethal mutations in the gene encoding elongation factor G of Escherichia coli. *J. Bacteriol.*, **176**, 123–9.
4. Lázár, V., Pal Singh, G., Spohn, R., et al. 2013, Bacterial evolution of antibiotic hypersensitivity. *Mol. Syst. Biol.*, **9**, 700.
5. Kohanski, M. A., Dwyer, D. J., Hayete, B., Lawrence, C. A., and Collins, J. J. 2007, A common mechanism of cellular death induced by bactericidal antibiotics. *Cell*, **130**, 797–810.
6. Mahoney, T. F., and Silhavy, T. J. 2013, The Cpx stress response confers resistance to some, but not all, bactericidal antibiotics. *J. Bacteriol.*, **195**, 1869–74.
